# Fructose regulates the pentose phosphate pathway and induces an inflammatory and resolution phenotype in Kupffer cells

**DOI:** 10.1038/s41598-024-54272-w

**Published:** 2024-02-18

**Authors:** Mareca Lodge, Grace Scheidemantle, Victoria R. Adams, Matthew A. Cottam, Daniel Richard, Denitra Breuer, Peter Thompson, Kritika Shrestha, Xiaojing Liu, Arion Kennedy

**Affiliations:** 1 Department of Molecular and Structural Biochemistry, NC State University, Raleigh, NC USA; 2 Department of Molecular Physiology and Biophysics, Vanderbilt University School of Medicine, Nashville, TN USA; 3 Molecular Education, Technology and Research Innovation Center (METRIC), NC State University, Raleigh, NC USA

**Keywords:** Carbohydrates, Cytokines, Lipidomics, Metabolomics, Hepatology, Endocrine system and metabolic diseases, Non-alcoholic fatty liver disease

## Abstract

Over-consumption of fructose in adults and children has been linked to increased risk of non-alcoholic fatty liver disease (NAFLD). Recent studies have highlighted the effect of fructose on liver inflammation, fibrosis, and immune cell activation. However, little work summarizes the direct impact of fructose on macrophage infiltration, phenotype, and function within the liver. We demonstrate that chronic fructose diet decreased Kupffer cell populations while increasing transitioning monocytes. In addition, fructose increased fibrotic gene expression of collagen 1 alpha 1 (*Col1a1)* and tissue metallopeptidase inhibitor 1 (*Timp1)* as well as inflammatory gene expression of tumor necrosis factor alpha (*Tnfa)* and expression of transmembrane glycoprotein NMB (*Gpnmb)* in liver tissue compared to glucose and control diets. Single cell RNA sequencing (scRNAseq) revealed fructose elevated expression of matrix metallopeptidase 12 (*Mmp12)*, interleukin 1 receptor antagonist *(Il1rn),* and radical S-adenosyl methionine domain (*Rsad2)* in liver and hepatic macrophages. In vitro studies using IMKC and J774.1 cells demonstrated decreased viability when exposed to fructose. Additionally, fructose increased *Gpnmb*, *Tnfa*, *Mmp12*, *Il1rn*, and *Rsad2* in unpolarized IMKC. By mass spectrometry, C13 fructose tracing detected fructose metabolites in glycolysis and the pentose phosphate pathway (PPP). Inhibition of the PPP further increased fructose induced *Il6, Gpnmb*, *Mmp12*, *Il1rn*, and *Rsad2* in nonpolarized IMKC. Taken together, fructose decreases cell viability while upregulating resolution and anti-inflammatory associated genes in Kupffer cells.

## Introduction

In recent years, the monosaccharide fructose has become one of the most studied carbohydrates as consumption is more prevalent in everyday diets^1^. Fructose consumption has been linked to obesity related type 2 diabetes mellitus, non-alcoholic fatty liver disease, (NAFLD) and atherogenic dyslipidemia, becoming a greater risk for cardiac stroke and potentially death^2^. Prevalence of steatosis in NAFLD has reached 25% of the worldwide population with the more aggressive form of non-alcoholic steatohepatitis (NASH) reaching nearly 14%^2^. Given the prevalence of NAFLD, it is important to understand the mechanism of how fructose mediates inflammation and fibrosis progression. Important work has summarized the effects of fructose on hepatocyte function and de novo lipogenesis in the liver^3^. However, little is known about the impact of fructose on resident and infiltrating immune cell populations.

The liver is made of several macrophage populations ranging from residential Kupffer Cells (KC) to infiltrating bone marrow derived monocytes and macrophages. Monocytes and macrophages play a large role in maintaining tissue function and phenotype by scavenging for toxins and secreting cytokines necessary for tissue resolution^4^. KC and recruited macrophages have been shown to play a massive role in the progression of NAFLD as they often display a pro-inflammatory phenotype (M1) secreting cytokines interleukin 6 (IL-6), tumor necrosis factor α (Tnfα) and interleukin 1β (IL-1β)^5–7^. KC depletion prevents steatosis, hepatic insulin resistance and inflammation in rodents^8,9^. Residential or recruited macrophages may express a fluid phenotype, displaying both proinflammatory and anti-inflammatory properties. External stimuli such as lipopolysaccharide (LPS) or interferon gamma (IFNγ) activate toll-like receptors (TLR) response of macrophages to secrete cytokines such as IL6 and TNFα to aid in tissue repair through increased flux of glycolysis^10^. Under chronic inflammatory conditions, macrophages may switch to an anti-inflammatory or resolution phenotype releasing extracellular matrix proteins to signal wound healing.

Carbohydrate metabolism is a key regulator of macrophage plasticity. Glucose is the primary energy source for macrophages. In contrast, limited studies address the impact of fructose metabolism on macrophage plasticity and the impact on NAFLD progression^11^. In LPS stimulated monocytes, fructose shifts glycolysis metabolism to the tri-carboxylic acid cycle (TCA) or oxidative phosphorylation (OXPHOS) cycling^12^. Furthermore, fructose increases secretion of pro-inflammatory cytokines IL-1β, IL6 and TNFα which are dependent on hexokinase activity. In dendritic cells, fructose undergoes glycolytic metabolism while activating nuclear factor kappa B (NFkB)^13^. M1 polarization is dependent on glycolysis. In addition, utilization of the PPP produces NADPH pools needed for cytokine production. Given the importance of KC in maintaining liver homeostasis, we investigate the role of fructose on regulating KC metabolism, inflammation, and viability using chronic-fed mouse models and immortalized KC in vitro.

In this study, fructose induced liver injury was associated with reduced KC and increased transitioning monocytes. Interestingly, macrophage populations from chronic fructose diet increased *Mmp12*, *Il1rn* and *Gpnmb* gene expression. Mass spectrometry revealed fructose carbon partitioning in IMKC through glycolysis and the PPP. Inhibition of the PPP led to elevated *Gpnmb*, *Il6*, *Mmp12*, *Il1rn* and *Rsad2* expression in fructose treated IMKC. Taken together these data indicate fructose drives a wound healing and anti-inflammatory phenotype in KC and reduces viability. The PPP may play an inhibitory role in regulating anti-inflammatory response in fructose exposed macrophages.

## Methods

All methods were performed in accordance with ARRIVE guidelines (https://arriveguidelines.org).

### Mouse model

Male C57BL/6J mice were housed at North Carolina State University Biological Resources Facility with ad libitum access to chow food (Laboratory Rodent Diet 5001) and water on a 12- hour light/dark cycle. At the age of 8 weeks, mice were administered 30% fructose, glucose, or no supplementation in their drinking water for 24 and 32 weeks. Chronic fructose diet data is combined from 24 and 32 weeks. Food and water intake was monitored every week as well as body weight. Before tissue collection mice were fasted for 5 h. Mice were then anesthetized with isoflurane and sacrificed via cervical dislocation. Liver was perfused by heart right ventricle puncture with 1X PBS. Liver and adipose tissue was removed and immediately snap-frozen in liquid nitrogen or fixed via formaldehyde. All animal procedures were approved by the Institutional Animal Care and Use Committee (IACUC) at North Carolina State University under protocol 21–502-B. Mice were handled and used strictly according to the guidelines of the American Veterinary Medical Association and Association for Assessment and Accreditation of Laboratory Animal Care. All methods were performed in accordance with the relevant guidelines and regulations.

### Liver nonparenchymal cell isolation

Isolated livers were immediately processed for nonparenchymal cell isolation using liver lymphocyte protocol adapted from Breuer et al. 2020 and Lynch et al. 2018^14,15^. Immune cells were separated by 443×*g* spins and 33% percoll gradient for 30 min. Samples were resuspended in FACS buffer and stained with propidium iodide and acridine orange for cell counting.

### Flow Cytometry

Liver nonparenchymal cells were incubated with Fc block and incubated in FACS buffer with fluorophore conjugated antibodies on ice for the following macrophage panel: F480 (A700, 1:200, Biolegend), CD64 (PE, 1:200, BD Biosciences), CD11b (FITC, 1:200, BD Biosciences), Ly6C (PerCP-Cy5.5, 1:200, BD Biosciences), CLEC4F (Unconjugated, 1:200, R&D Systems). Following antibody incubation samples were washed twice with FACS buffer. After washing, CLEC4F samples were incubated with a secondary antibody (Donkey anti-goat AF647, 1:500, ThermoFisher) and washed twice after incubation. The Becton Dickinson LSRII machine at the NCSU Flow Cytometry Core was used to acquire all flow data. Flow data was analyzed using FlowJo software v10.8.

### Magnetic labeling and separation

Magnetic separation was performed following the Miltenyi Biotec manufacturer's protocol. Remaining pellet and supernatant were counted and resuspended in buffer per 1 × 10^7^ total cells. Anti-F4/80, Anti-FITC and Anti-CD11b MicroBeads UltraPure was added to 1 × 10^7^ cells. Cells were incubated for 15 min in the dark at 2–8 °C. Cells were washed with FACS and centrifuged at 300×*g* for 10 min. Supernatant was removed and cells were resuspended in FACS buffer.

### Single cell RNA sequencing

All samples were submitted and processed by the NCSU Genomics Core. Sample preparation was conducted using the 5′ assay for the 10X Chromium Controller (10X Genomics) targeting 5,000 cells per sample. In all, 50,000 reads per cell were targeted for PE-150 sequencing on an Illumina NovaSeq. Sample processing and sequencing were completed in the NCSU Genomics Sciences Laboratory. Reads were further processed using CellRanger v4.1 (10X Genomics) with the mm9 mouse reference genome. Ambient RNA signal was estimated and removed using SoupX v1.5.2 with the autoEstCont and adjustCounts functions. Filtered count matrices were passed to Seurat v4 and cells with < 10% mitochondrial RNA content and at least 500 unique reads that correspond to at least 200 features were retained^16^. DoubletFinder v2.0.3 was used to detect and remove heterotypic doublets^17^. Counts were normalized using the NormalizeData function from Seurat.

### Data Integration and dimensional reduction

Principal component analysis was performed using the RunPCA function from Seurat. Integration anchors were detected using the FindIntegrationAnchors function with reduction = “rpca” and independent samples were integrated with the IntegrateData function from Seurat. Nonlinear dimensional reduction was performed using the RunUMAP function from Seurat using the first 30 principal components.

### Unsupervised clustering and cell annotation

Unsupervised clustering was performed using the Louvain algorithm implemented in the FindClusters function with resolution = 0.4 in Seurat. Cell types were predicted using SingleR v1.6.1 with the Immgen and MouseRNAseq references from the celldex v1.2.0 package^18^. Consensus between predicted cell type labels, differentially expressed markers found using the FindAllMarkers function, and established cell-specific markers were used to annotate clusters. For each major cell type, subclustering was performed to further delineate cell subtypes.

### Downstream single cell analysis

For assessment of differential abundance between conditions, MiloR v1.6.0 was used to generate neighborhoods of cells and comparisons were performed using the testNhoods function^19^. For additional plotting, the integrated Seurat v4 object was converted to an anndata object using scDIOR^20^. Then, anndata objects were loaded into Scanpy v1.9.2^21^.

### Liver tissue histology staining

Paraffin-embedded sections from mouse livers were used for hematoxylin and eosin (H&E) and Sirius red staining^14^. Whole slide images were captured on BioTek Cytation 5 at 10X magnification and quantified by ImageJ Software.

### RNA isolation and RT-PCR

Samples were isolated for RNA according to the manufacturer's protocol using Direct-zol™ RNA MicroPrep kit (Genesee Scientific). RNA concentration was obtained by nanodrop, and cDNA was made using qscript cDNA supermix following the manufacturer’s instructions (Quantabio). cDNA was synthesized at 1000 ng using BioRAD iQ5 thermocycler. Cycles were: Priming for 5 min at 25 °C, RT: 30 min at 42 °C and RT inactivation for 5 min at 85 °C. cDNA was used to analyze gene expression by real time polymerase chain reaction (RT q-PCR) using PerfeCTa qPCR FastMixII (QUantbio). TaqMan assays used were *Il6*, *Tnfα*, *Il1b*, *Gpnmb*, *Col1a1*, *Timp1*, *Mmp12*, *Rsad2*, *Ilrn1*, *Il1bp* and 18S eukaryotic endogenous control (Thermo Fisher Scientific). Samples were normalized to 18S.

### Sample preparation for LC–MS analysis

IMKC, RAW 264.7, and J774A.1 cells were seeded at 5 × 10^6^ cells/ml in 6 well plates in RPMI culturing media. 24 h later media was changed to C13 labeled 5 mM or 25 mM glucose or C13 labeled 5 mM or 25 mM fructose. After 24 h, cells were washed twice with ice cold saline (0.9% NaCl) and immediately placed on dry ice. Extraction solution composed of methanol/water (4:1, v/v) was added to each well and cells were scraped, vortexed vigorously and spun down at 20,000 × g for 10 min at 4 °C. Supernatant containing polar metabolites were dried in vacuum concentrator. Dry pellets and cell media were kept frozen at -80 °C until ready for LC–MS analysis.

### LC–MS analysis

Metabolite analysis was adapted from Duan et al. 2022^22^ performed using Ultimate 3000 UHPLC (Dionex) coupled with Q Exactive Plus mass spectrometer (Thermo Fisher Scientific) and Vanquish UHPLC (Thermo Fisher Scientific) coupled with an Orbitrap Exploris 480 mass spectrometer (Thermo Fisher Scientific), as described previously. A hydrophilic interaction chromatography method (HILIC) with an Xbridge amide column (100 × 2.1 mm i.d., 3.5 μm; Waters) was used for metabolite separation at 25 °C. Mobile phase A: water with 5 mM ammonium acetate (pH 6.8), and mobile phase B: 100% acetonitrile. Linear gradient is: 0 min, 85% B; 1.5 min, 85% B; 5.5 min, 35% B; 6.9 min, 35% B; 10.5 min, 35% B; 10.6 min, 10% B; 12.5 min, 10% B; 13.5 min, 85% B; 17.9 min, 85% B; 18 min, 85% B; 20 min, 85% B. Due to the instrumentation difference between Ultimate 3000 UHPLC and Vanquish UHPLC, different flow rates were used. For Ultimate 3000 UHPLC, the flow rate is: 0–5.5 min, 0.15 ml/min; 6.9–10.5 min, 0.17 ml/min; 10.6–17.9 min, 0.3 ml/min; 18–20 min, 0.15 ml/min. For Vanquish UHPLC, the flow rate is: 0–5.5 min, 0.11 ml/min; 6.9–10.5 min, 0.13 ml/min; 10.6–17.9 min, 0.25 ml/min; 18–20 min, 0.11 ml/min. Both mass spectrometers were equipped with a HESI probe and operated in the positive/negative switching mode. When Q Exactive Plus mass spectrometer was used, the relevant parameters are as listed: heater temperature, 120 °C; sheath gas, 30; auxiliary gas, 10; sweep gas, 3; spray voltage, 3.6 kV for positive mode and 2.5 kV for negative mode; capillary temperature, 320 °C; S-lens, 55. The resolution was set at 70,000 (at *m/z* 200). Maximum injection time (max IT) was set at 200 ms and automatic gain control (AGC) was set at 3 × 10^6^. When Exploris 480 mass spectrometer was used, the relevant parameters are as listed: vaporizer temperature, 350 °C; ion transfer tube temperature, 300 °C; sheath gas, 35; auxiliary gas, 7; sweep gas, 1; spray voltage, 3.5 kV for positive mode and 2.5 kV for negative mode; RF-lens (%), 30. The resolution was set at 60,000 (at *m/z* 200). Automatic maximum injection time (max IT) and automatic gain control (AGC) were used.

Samples were reconstituted into sample solvent (40 μl water:ACN:MeOH (2:1:1, v/v/v) and 3 μl was injected to LC–MS for polar metabolite analysis. Metabolite identification was based on exact mass to charge ratio (less than 5 ppm) and retention time, which was determined using an in-house library. Unresolved metabolites were combined into one feature to reflect the possibility of co-eluted isomers. Integrated peak area of each metabolite was used to calculate the relative changes of metabolites in different samples. Integrated peak area of each metabolite or each isotopologue was used to calculate the ^13^C enrichment without natural abundance correction.

### Cell culture

IMKC and RAW 264.7 cells were cultured in RPMI (VWR 1640 contains 11 mM glucose) with 10% fetal bovine serum (FBS), 5% L-Glutamine, 1% Penicillin/Streptomycin. All cells were cultured for 24 h at 37 °C with 5% CO2 in the atmosphere before treatment and seeding at 4 × 10^5^ cells/ml. Cells were treated with RPMI with no glucose and 5 or 25 mM glucose or fructose was added in the absence (M0 polarized macrophages) or presence of 0.1ug/ml LPS (M1 polarized macrophages) (Fisher Scientific 50–112-2025). Cells were harvested 24 h after treatment for protein, RNA, or media collection. Protein was harvested by washing once with 1X PBS and adding RIPA buffer (150 mM Sodium Chloride, 1% Triton X, 0.5% Sodium Deoxycholate, 0.1% SDS, 50 mM Tris, and 1 mM EDTA) with protease inhibitor cocktail 1:100 (HALT). Plates were harvested for RNA by Tri-reagent (Fisher Scientific). Media was collected and stored at -80 °C.

### BrdU assay

IMKC were plated in 96 well plates with a seeding density of 2.5 × 10^4^ cells/well in culturing RPMI. Cells were then treated with 25 mM glucose or fructose for 24 h. BrdU reagent was added at time of treatment using the BrdU Assay Kit (#6813 Cell signaling technology) and absorbance measured at 450 nm on plate reader.

### MTT assay

IMKC were plated in 96 well plates with a seeding density of 2.5 × 10^4^ cells/well in culturing RPMI and adhered overnight. Culturing media was removed and replaced with glucose or fructose media (RPMI 1640) without phenol red with 1.2 mM MTT labeling reagent. Cells were incubated at 37 °C for 2 h. Cells were then incubated at 37 °C for 10 min and absorbance measured at 450 nm on plate reader.

### Western blotting

Protein samples were thawed and spun at 15,000 RPM at 4 °C. Supernatant was used for BCA protein assay kit (Thermo Scientific) to quantify concentration of protein. Samples were prepared using 2X Laemmli Sample Buffer (Bio-rad) in a 1:1 ratio of protein to loading buffer solution. Samples were run on 4–15% 12 well SDS page gel. The gel was transferred for 1 h and blocked using the One-block blocking buffer (Genesee Scientific) for 1 h. Glucose 6 phosphate dehydrogenase (G6PDH), Hexokinase 1 and β-actin antibodies were purchased from cell signaling. Glutamic–pyruvic transaminase 2 (GPT2) was purchased from sigma. All antibodies were used at 1:1000 dilutions except β-actin at 1:3000 dilution. Primary antibodies were incubated for 1 h at room temperature or overnight at 4 °C shaking. The membrane was washed with PBS with 1% TWEEN-20 for 5 min, then incubated with secondary antibodies goat anti-mouse and goat anti-rabbit (Fisher Scientific) at a 1:20,000 dilution. The secondary was removed, and the membrane was washed with PBS with 1% TWEEN-20 for 5 min. Membrane bands were analyzed by Li-COR Odyssey and quantified by Image Lite Version 5.2 software.

### G6PDH inhibition studies

IMKC cells were plated in culturing media at 4 × 10^5^ cells/ml in 24 well plates and adhered overnight. Cells were treated as described above with or without the G6PDH inhibitor 6 Aminonicotinamide (6AN) (Milipore Sigma-CAS: 329-89-5) at 100 µM for 24 h. J774.1 cells were treated with 50 µM Dehydroepiandrosterone for 24 h. Cells were harvested for RNA. G6PDH knock down was established using Invitrogen™ Lipofectamine™ RNAiMAX Transfection Reagent (Fisher Scientific-13–778-030) and Silencer® Select Pre-Designed & Validated siRN AID s66340 (Thermo Scientific-4390771). siRNA studies were optimized to 25 pmol of siRNA for 48 h. IMKC were plated at 2 × 10^5^ cells/ml in 24 well plates for siRNA studies. The next day media was changed to fresh culture media, or fresh culture media with 25 pmol siRNA. 24 h later media was changed again to fresh culturing media as a control, 25 mM glucose or fructose. Cells were then polarized to M0 or M1 with or without additional 25 pmol of G6PDH siRNA. After 24 h of treatment samples were collected for RNA and protein.

### ELISA

IL6 ELISA kit was purchased from Invitrogen (Thermo Fisher BMS6032) and protocol was followed per kit recommendations. Media was spun at 500×*g* for 10 min to pellet cell debris before being added to wells.

### Apoptosis assay

IMKC cells were plated at 2 × 10^4^ cells/ well in a 96 black walled plate. Cells were treated with glucose or fructose RPMI media for 24 h. Cells were tested for viability, cytotoxicity and apoptotic activity following ApoTox-Glo Triplex Assay (G6320 Promega) manufacturers protocol.

### Statistical analysis

GraphPad Prism 9.3.1 software was used for all statistical analyses. Two-tailed unpaired Student’s t-tests were performed for two group comparisons. Two-way ANOVA was performed for genotype versus diet studies followed by multiple T test comparisons. All data is presented as the mean ± SEM. Data was considered statistically significant for *P < *0.05 (*), *P < *0.01 (**), *P < *0.001(***), and *P < *0.0001(****).

## Results

### Fructose induces liver injury

**Figure 1:**
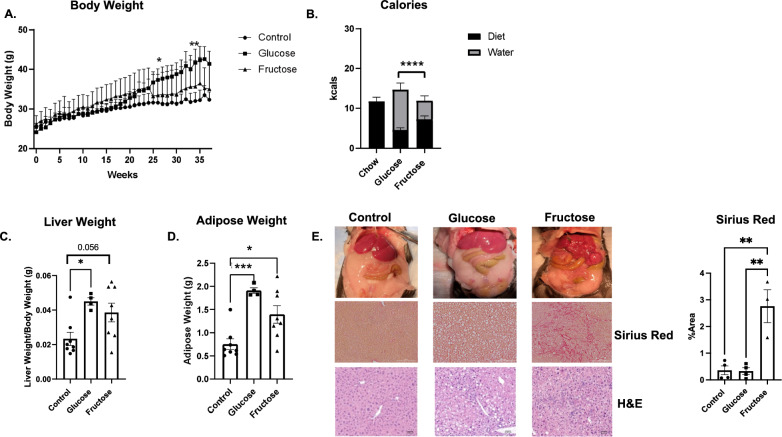
Fructose Induces Liver and Adipose Tissue Weight Gain. C57BL/6 mice were fed chow diet supplemented with 30% glucose, fructose or control water for 32 weeks. (**A)** Body weight gain over time. **(B)** Chow and water calorie intake over 32 weeks, water consumption analyzed between groups. **(C,D)** Liver and gonadal adipose tissue were removed and weighed at 32 weeks**. (E)** Liver morphology and histology using H + E and Sirius red. * *P < *0.05 ** *P < *0.01, *** *P < *0.001, **** *P < *0.0001. Data are representative of two independent experiments and show mean ± SEMs control (n = 8), Glucose (n = 4) and Fructose (n = 8).

**Figure 2:**
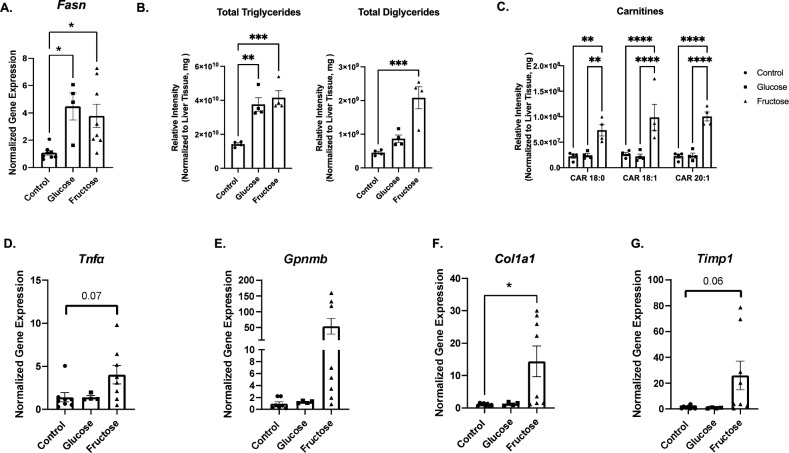
Fructose Increases Long Chain Carnitines and DGs and Inflammation. Liver tissue was isolated and processed for RT qPCR and mass spectrometry lipidomics analysis. **(A)** Gene expression of lipid synthesis gene *Fasn*. Intensity of long chain fatty acid acyl carnitines **(B)** and diglycerides **(C)** normalized to liver weight, from mass spectrometry profiling. Livers were isolated and processed for RT qPCR and analyzed for inflammatory gene Tnfα **(D),** anti-inflammatory gene *Gpnmb* **(E)** and fibrotic genes *Col1a1(F)* and *Timp1* **(G)**. * *P < *0.05, ** *P < *0.01, *** *P < *0.001, **** *P < *0.0001. Data are representative of two independent experiments and show mean ± SEMs Control (n = 8), Glucose (n = 4) and Fructose (n = 8).

To gain insight into the impact of fructose exposure on liver pathology, mice were fed chow diet supplemented with control water, 30% glucose water or 30% fructose water for 32 weeks. Mice on glucose or fructose water had elevated body weight compared to control (Fig. 1A). Interestingly, mice on fructose had decreased weight gain compared to glucose even when consuming a similar number of combined calories from water and diet (Fig. 1B). Fructose supplementation for 16 weeks increased liver and adipose weight compared to control (Supplemental Fig. 1A,B). Fructose liver weight was significantly increased in fructose fed mice compared to glucose at 32 weeks of diet (Fig. 1C). In addition, adipose tissue weight was elevated at 32 weeks of fructose supplementation compared to control (Fig. 1D). Fructose is known to increase de novo lipogenesis leading to increased lipid accumulation and stored triglycerides. We confirmed fructose elevated fatty acid synthase (*fasn)* gene expression at 16 and 32 weeks (Supplemental Fig. 1C, Fig. 2A). Targeted mass spectrometry lipidomics profiling demonstrated fructose significantly increased total triglycerides and diglycerides (Fig. 2B) and saturated long chain fatty acid acyl-carnitines, specifically 18:0, 18:1 and 20:1 compared to control and glucose fed mice (Fig. 2C). Interestingly, at 16 weeks of fructose administration, only total diglycerides were significantly increased compared to controls (Supplemental Fig. 1G). Fructose is associated with increased inflammation and fibrosis which may be due to cellular crosstalk and cytokine secretion^23^. With increased inflammation, cellular response may include upregulation of anti-inflammatory cytokines to suppress inflammation. Recently, GPNMB has been reported to protect the liver from liver fat toxicity and is highly expressed in NASH associated lipid laden macrophages^24^. Fructose diet increased *Tnfa* and *Gpnmb* hepatic gene expression (Fig. 2D,E). In addition, fructose significantly increased fibrosis related genes *Col1a1* and *Timp1* compared to control (Fig. 2F,G). Interestingly, at 16 weeks of fructose supplementation no changes in inflammation and fibrosis associated genes were detected (Supplemental Fig. 1D,E,F). Fructose supplementation also significantly increased liver injury measured by liver GPT2 protein levels at 16 and 32 weeks (Supplemental Fig. 1H) and Sirius red staining at 32 weeks which measures collagen (Fig. 1E). Additionally, fructose fed mice displayed oval cell hyperplasia, hepatocyte damage, and tumor nodules compared to control and glucose fed mice (Fig. 1E). These data demonstrate chronic fructose exposure increases hepatic inflammatory response and fibrosis.

### Fructose decreases KC and increases transitioning monocytes

**Figure 3:**
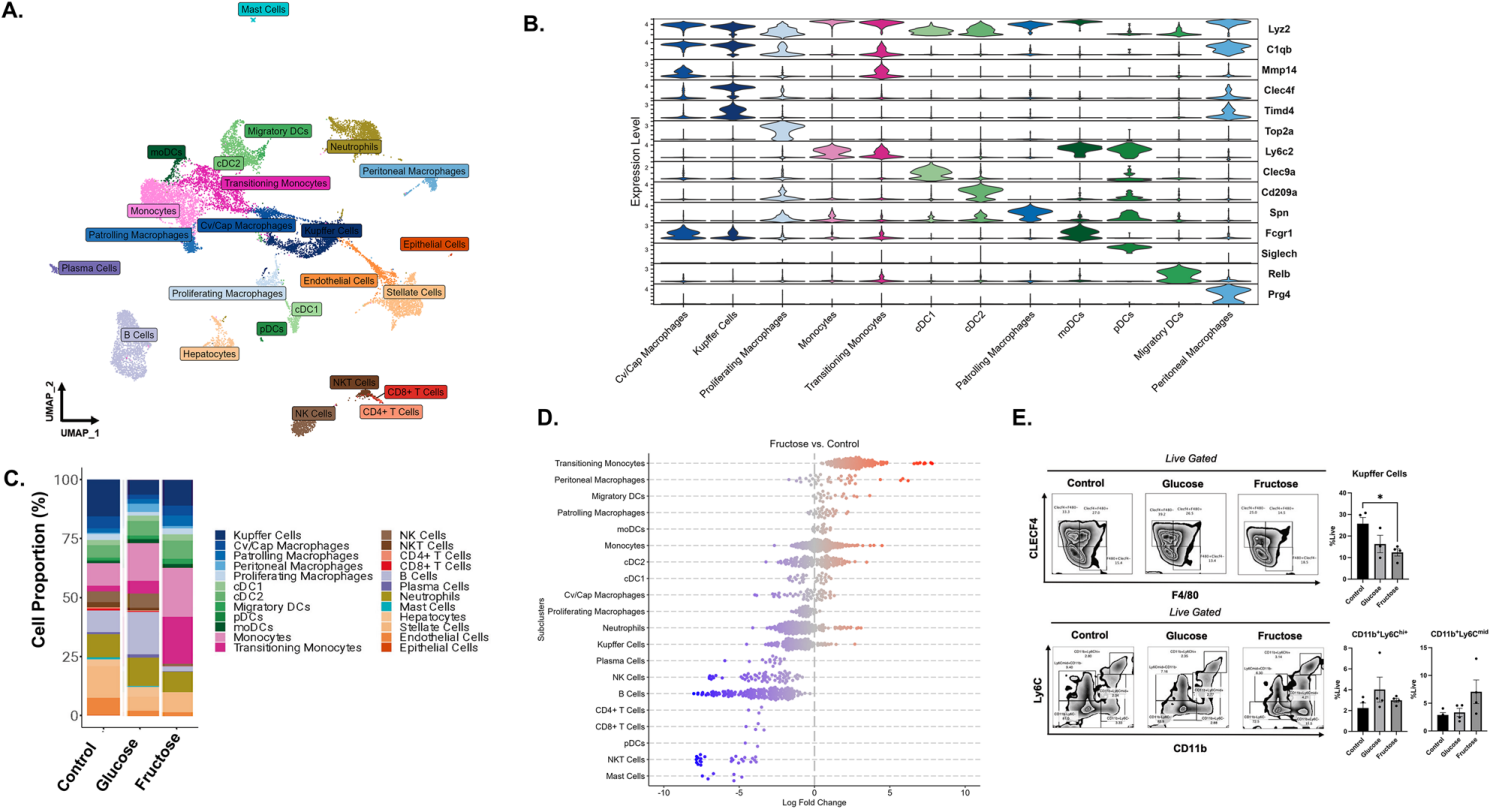
Fructose Decreases Kupffer Cells and Increases Transitioning Monocytes. Livers of fructose, glucose, and control mouse fed for 32 weeks were collected and Cd11b^+^F4/80^+^ cells were isolated and scRNAseq analysis. Control (n = 4), Glucose (n = 4) and Fructose (n = 4). **(A)** Unbiased clustering of single cells labeled by cell type category and colored by high-resolution cell type identities by UMAP. **(B)** Violin plot of select cluster markers for myeloid cells. **(C)** scRNAseq analysis of cell proportions in control, glucose, and fructose fed mice. **(D)** Neighborhoods of cells calculated using miloR of fructose vs. control. Each dot represents one cell. Blue decreased in fructose and red increased in fructose compared to control. **(E)** Flow analysis and quantification of Kupffer cells (Clecf4^+^F4/80^+^), infiltrating monocytes (Cd11b^+^Ly6C^hi^) and CD11b^+^Ly6C^mid^ cells. Data are representative of two independent experiments and show mean ± SEMs (n = 4).

To determine the potential mechanism of fructose induced liver injury, we analyzed the gene signature of hepatic macrophage subsets. Fructose diet has been demonstrated to increase KC, infiltrating monocytes, and macrophages, within the liver compared to control diets^25,26^. We used single cell RNA sequencing (scRNAseq) to determine how fructose alters hepatic monocyte and macrophage subsets (Fig. 3A). Fructose treatment decreased KC expressing *Ly6z2*, *C1qb*, *Clecf4*, *Timd4*, *Fcgr1* and B cells expressing *Cd24a*, *Cd79a*, *Ms4a1*, and *Mzb1*. In contrast, transitioning monocytes expressing *Lyz2*, *Ccr2*, *C1qb*, *Mmp14*, and *Ly6c2* increased with fructose diet compared to control and glucose (Fig. 3B–D and Supplemental Fig. 3). To confirm these findings, flow cytometry analysis identified significant reductions in KC (*F480*^+^*CD64*^*-*^*CLEC4F*^+^) populations in fructose treated mice compared to control and glucose (Fig. 3E). However, CD11b^+^Ly6C^hi^ monocyte populations were not significantly different between diets (Fig. 3E).

**Figure 4:**
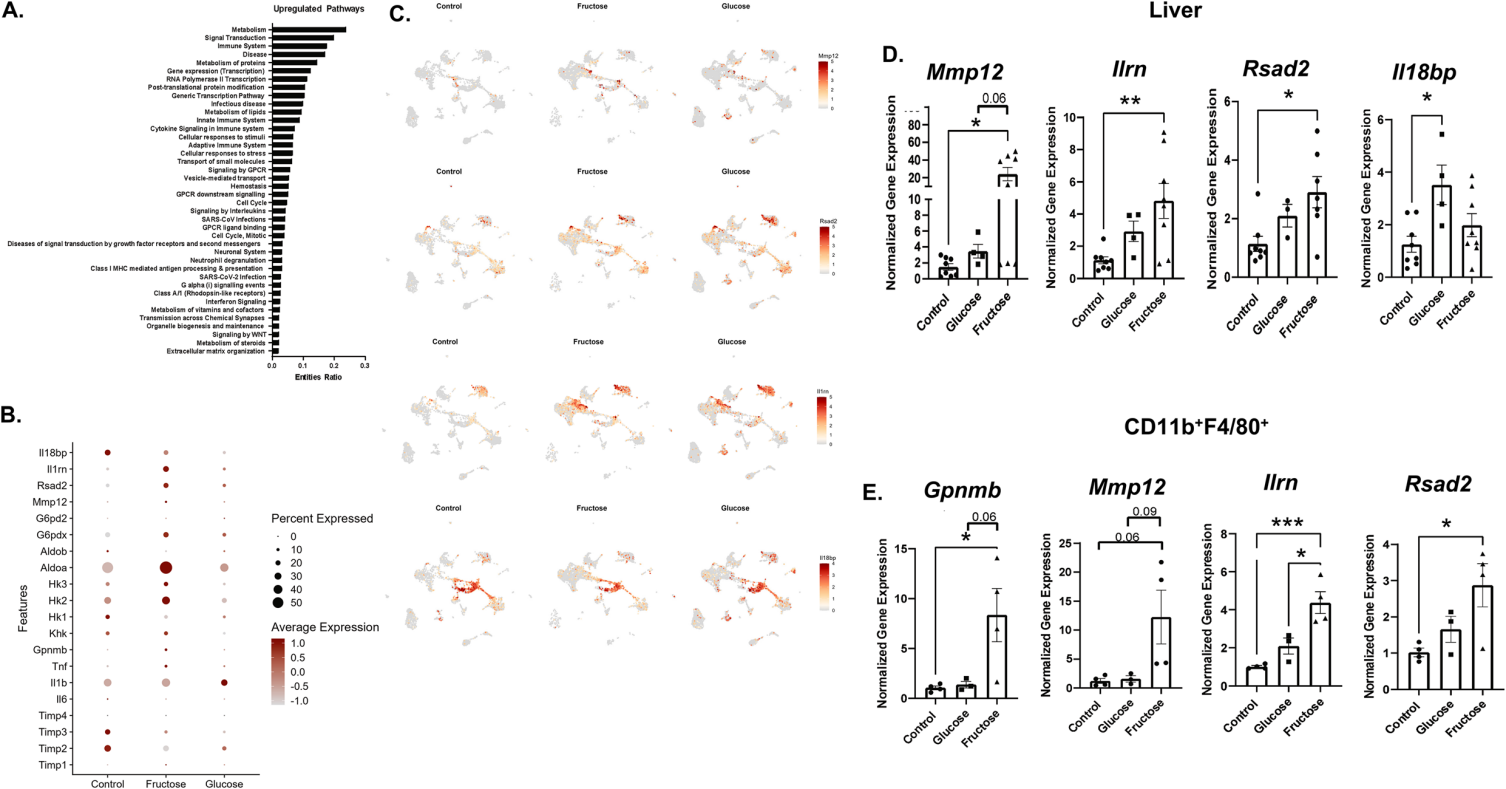
Fructose Increases Resolution and Anti-Inflammatory Gene Expression of Liver and Hepatic Macrophages. Hepatic Cd11b^+^ F4/80^+^ cells isolated from livers of fructose, glucose, and control mouse fed for 32 weeks. **(A)** Metascape pathway analysis of upregulated genes identified from scRNAseq in fructose fed mice. (**B,C**) scRNAseq data was analyzed for significantly upregulated genes in fructose fed mice compared to glucose and control. Livers were harvested and processed for RT qPCR as well as sorted into Cd11b^+^F4/80^+^ populations for RT-PCR. **(D)** Liver tissue resolution and anti-inflammatory genes *Mmp12*, *Il1rn*, *Rsad2* and *I18bp*. Data are representative of two independent experiments and show mean ± SEMs Control (n = 8), Glucose (n = 4). and Fructose (n = 8) **(E)** Cd11b^+^F4/80^+^ cell fractions analyzed by RT qPCR for genes *Mmp12*, *Il1rn, Rsad2* and *Gpnmb*. Data show mean ± SEMs Control (n = 4), Glucose (n = 4), Fructose (n = 4). * *P < *0.05, *** *P < *0.001.

scRNAseq of KC revealed fructose diet upregulated pathways involved in metabolism, signal transduction, and the immune system (Fig. 4A). By examining scRNAseq genes by treatment, the inflammatory gene *Tnfa* and anti-inflammatory *Gpnmb* were elevated in fructose conditions (Fig. 4B). In addition, genes involved in fructose metabolism, *Hk2, Hk3* and *Aldoa,* were also increased. Lastly, *G6pdx*, the rate limiting step of the PPP, had a higher frequency in fructose fed mice compared to control and glucose (Fig. 4B). While the frequency was higher under fructose conditions, these genes were not found to be differentially regulated in KC. *Mmp12*, *Il1rn*, *Rsad2,* and *Il18bp* which are associated with wound healing, resolution, and anti-inflammation were upregulated in KC from fructose fed mice (Fig. 4C).

To confirm the scRNAseq data, gene signatures of liver tissue and isolated Cd11b^+^F4/80^+^ macrophages were analyzed. In liver tissue, *Mmp12*, *Il1rn* and *Rsad2* were significantly increased in fructose treated mice compared to control water (Fig. 4D). Fructose had no significant impact on *Il18bp* expression. Fructose also significantly increased *Gpnmb*, *Mmp12*, *Il1rn*, and *Rsad2* in isolated CD11b^+^F4/80^+^ cells isolated from livers of mice (Fig. 4E). These findings suggest fructose directly regulates anti-inflammatory and wound healing gene expression in hepatic macrophages.

### Fructose reduces metabolic activity and cell viability of IMKC

**Figure 5:**
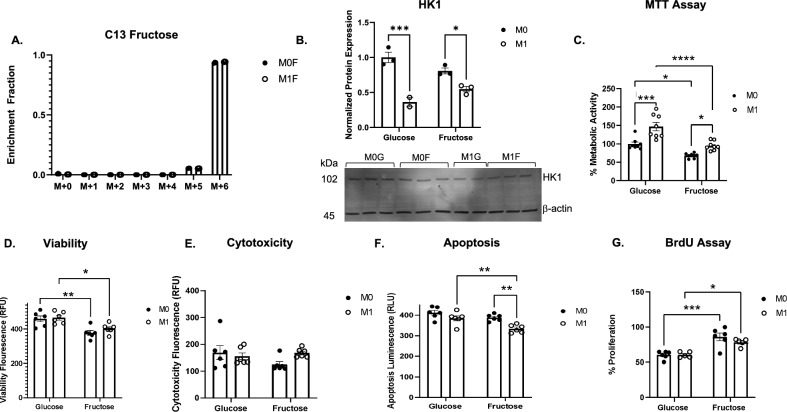
Fructose Uptake Decreases Cell Viability in IMKC. IMKC were treated with C13 25 mM glucose or fructose for 24-h with (M1) or without (M0) 0.1ug/ml LPS. **(A)** Cells were harvested for MS using MetOH extraction for metabolite analysis and analyzed by MS peak intensity relative to total fructose in IMKC (n = 3). **(B)** Hexokinase 1 protein expression measured by western blot analysis (n = 3). The original blot is presented in Supplementary Fig. 5 (**C)** Cell viability measured by MTT labeling of cells (n = 8). **(D-F)** Viability assay measured at 400/505 nm, cytotoxicity measured at 485/520 nm. Measured luminescence for apoptosis assay (n = 6). **(G)** Cells were plated with BrdU proliferation assay and treated for 24 h (n = 6). Data are representative of three independent experiments and show mean ± SEMs. * *P < *0.05, ** *P < *0.01, *** *P < *0.001, **** *P < *0.0001.

Flow analysis and scRNAseq datasets revealed fructose reduced KC populations and increased resolution and anti-inflammatory gene expression. We sought to determine if fructose directly regulates KC function in vitro using three cell lines, IMKC, RAW 264.7 and J774.1 cells. Fructose uptake was analyzed via mass spectrometry in nonactivated (M0) and LPS stimulated (M1) macrophages treated with 25 mM fructose for 24 h. C13 labeled fructose peaks were observed after 24 h in IMKC (Fig. 5A), J774.1 and RAW 264.7 cell lines (Supplemental Fig. 4A,B). To identify enzymes responsible for fructose metabolism, we measured protein expression of hexokinase 1 (HK1) which phosphorylates fructose or glucose. HK1 expression did not differ between glucose and fructose treatment, however there was a significant decrease in HK1 expression in M1 polarized IMKC (Fig. 5B). Within the liver, fructose is phosphorylated by ketohexokinase (KHK), however KHK was not detected in IMKC, J774.1 or RAW 264.7 cells.

To examine the effect of fructose uptake on IMKC and J774.1 metabolic activity, we conducted an NADPH dependent MTT assay. The MTT assay measures cellular metabolic activity, viability, and proliferation^27^. Fructose significantly reduced metabolic activity in M0 and M1 IMKC (Fig. 5C) and J774.1 cells (Supplemental Fig. 4C) compared to glucose. To confirm fructose reduced cell viability, an alternative method was used, the ApoTox-Glo Triplex assay, to measure cell viability, cytotoxicity, and apoptosis. Similar to the MTT assay, fructose significantly reduced cell viability in M0 and M1 IMKC and J774.1 cells compared to glucose (Fig. 5D, Supplemental Fig. 4D). Interestingly, fructose had no impact on cytotoxicity in M0 and M1 IMKC (Fig. 5E). In contrast, fructose significantly increased cytotoxicity in M0 and M1 J774.1 cells compared to glucose (Supplemental Fig. 4E). In addition, fructose reduced apoptosis in M1 IMKC (Fig. 5F). However, fructose significantly increased apoptosis in M0 and M1 J774.1 cells (Supplemental Fig. 4F). Glucose is known to facilitate macrophage proliferation. However, it is unknown how fructose regulates macrophage proliferation^28^. Based on our data demonstrating that fructose reduces cell viability and has no impact on cell death in IMKC, we investigated the impact of fructose on KC proliferation. Using the BrdU assay, fructose significantly increased the proliferation of both M0 and M1 IMKC (Fig. 5G) while decreasing proliferation in J774.1 cells (Supplemental Fig. 4G). Taken together, fructose reduces cell viability in macrophages but does not lead to cytotoxicity or apoptosis in IMKC. Interestingly, fructose increases the proliferation of IMKC.

### Fructose metabolism induces anti-inflammatory and wound healing phenotype in IMKC

**Figure 6:**
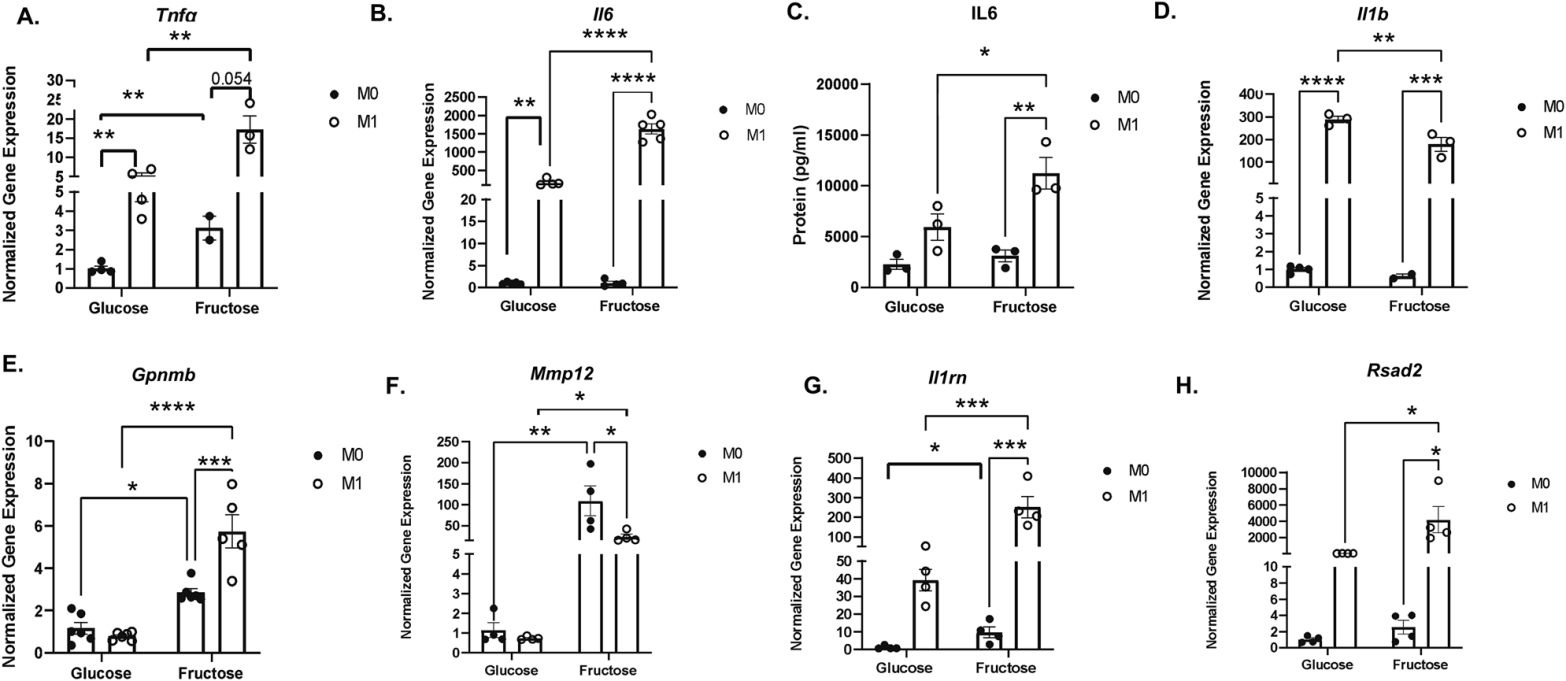
Fructose Upregulates Anti- and Pro-Inflammatory Gene Expression in IMKC. IMKC were treated with 25 mM glucose or fructose for 24-h without (M0) or with (M1) 0.1ug/ml LPS. RT-PCR analysis and gene expression of **(A)** *Tnfα,* **(B)** *Il6, (C)* ELISA analysis of IL6 protein levels in the media, and ***(D) Il-1b*** gene expression. Anti-inflammatory and resolution gene expression of **(E)** *Gpnmb*, **(F)** *Mmp12*, **(G)** *Il1rn,* and ***(H)*** *Rsad2*. Data are representative of three independent experiments and show mean ± SEMs (n = 4). * *P < *0.05, ** *P < *0.01, *** *P < *0.001, **** *P < *0.0001.

From scRNAseq analysis, chronic fructose diet induces a resolution and anti-inflammatory phenotype in KC. However, it is unknown if fructose directly or indirectly drives the KC phenotype. We investigated the direct impact of fructose on the genetic signature of macrophages. Fructose significantly upregulated the inflammatory cytokine *Tnfα* gene expression compared to glucose in M0 IMKC (Fig. 6A). In contrast, fructose did not induce *Il6* and *Il1b* gene expression in M0 IMKC, RAW 264.7 or J774.1 (Fig. 6B,D, Supplemental Fig. 6B,C,F,H respectively), or IL-6 protein levels (Fig. 6C and Supplemental Fig. 6G). Fructose also increased *Gpnmb, Mmp12* and *Il1rn* compared to glucose in M0 IMKC and increased *Gpnmb* in M0 J774.1 cells (Fig. 6E–G and Supplemental Fig. 6I).

In M1 polarized macrophages, fructose significantly increased *Tnfα* and *Il6* gene expression as well as protein secretion of IL6 in IMKC (Fig. 6A–C) compared to glucose. *Tnfa* gene expression was increased in both M1 RAW 264.7 and J774.1 cells (Supplemental Fig. 6A,E). However, *Il6* remained unchanged in M1 RAW 264.7 cells while gene and protein expression were decreased in M1 J774.1 cells (Supplemental Fig. 6B,F,). Interestingly, fructose reduced expression of *Il1b* in M1 IMKC, RAW 264.7 and J774.1 cells (Fig. 6D, Supplemental Fig. 6C,H respectively). Gene expression of *Gpnmb* was increased in M1 macrophages (Fig. 6E, Supplemental Fig. 6D,I). Interestingly, *Mmp12*, *Il1rn* and *Rsad2* were upregulated in M1 IMKC (Fig. 6F–H). These results indicate fructose directly regulates extracellular matrix and anti-inflammatory associated genes in unpolarized and polarized KC.

### C13 fructose labeling reveals fructose PPP carbon shuttling

**Figure 7:**
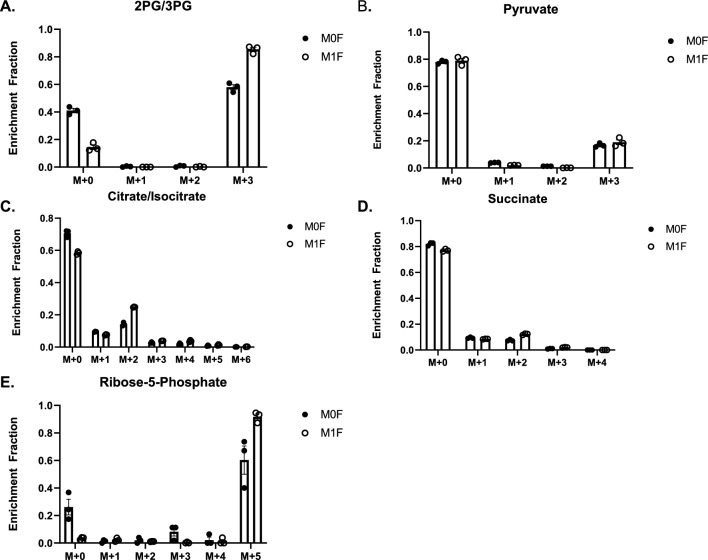
Fructose Metabolism Partitioning in IMKC. IMKC were plated in 25 mM glucose for 24 h before media was changed to C13 25 mM fructose without (M0) or with (M1) 0.1ug/ml LPS for 24 h and harvested for LC/MS by MetOH extraction for metabolite analysis and analyzed by MS peak intensity. Metabolites of interest **(A,B)** glycolysis intermediates, **(C,D)** TCA cycle intermediates, **(E)** PPP intermediates**.** Metabolite tracing. Data are representative of three independent experiments and show mean ± SEMs (n = 3).

To understand how fructose impacts macrophage and KC metabolism, IMKC, RAW 264.7 and J774.1 cells were treated with C13 labeled fructose for 24 h. Jones et al. recently showed 11.1 mM fructose treated monocytes had increased carbon shuttling through glycolysis and the TCA cycle^12^. In comparison, we found glycolysis intermediates 2/3- phosphoglycerate, pyruvate, dihydroxyacetone phosphate, and fructose 1,6 bisphosphate fructose carbons utilized by the glycolysis pathway in IMKC (Fig. 7A-B), RAW 264.7 and J774.1 cells (Supplemental Fig. 7A,B,E,F respectively). In contrast, TCA intermediates were not detected under fructose conditions in any macrophage cell lines (Fig. 7C-D, Supplemental Fig. 7C,G–H). However, C13 labeled fructose carbons were detected in PPP intermediates, specifically ribose 5-phosphate phosphate (Fig. 7E, Supplemental Fig. 7D,I). These findings suggest KC metabolize fructose through glycolysis and the PPP with limited shuttling into the TCA cycle.

### Pentose phosphate pathway regulates fructose mediated inflammatory gene expression

We next analyzed the role of the PPP in fructose stimulated gene expression in KC. We targeted G6PDH, the rate limiting enzyme in the PPP, using the competitive inhibitor 6AN and noncompetitive inhibitor DHEA^29^. Inhibition of the PPP augmented expression of fructose induced *Il6* gene expression in both M0 IMKC and J774.1, although there was no difference in *Tnfα* expression compared to fructose (Supplemental Figs. 8A,B and 9A,B). In M0 IMKC, 6AN further increased fructose induced expression of *Gpnmb* and *Rsad2* (Supplemental Fig. 8C,F).

To evaluate the effect of PPP inhibition on fructose induced gene expression of M1 polarization we treated macrophages with or without LPS and 6AN in fructose conditions. Although inhibition of the PPP increased *Il6* expression in both M0 IMKC and J774.1, fructose decreased *Il6* expression in M1 IMKC and increased expression in M1 J774.1 cells (Supplemental Fig. 8A, 9A). Unique to M1 J774.1 cells, DHEA decreased *Tnfα* expression (Supplemental Fig. 8B). Expression of fibrotic gene *Timp1* was decreased in M1 DHEA J774.1 (Supplemental Fig. 9C). 6AN inhibition of the PPP also decreased *Gpnmb*, *Il1rn* and *Rsad2* in M1 IMKC, while increasing the expression of *Mmp12* (Supplemental Fig. 8C, E–F).

**Figure 8:**
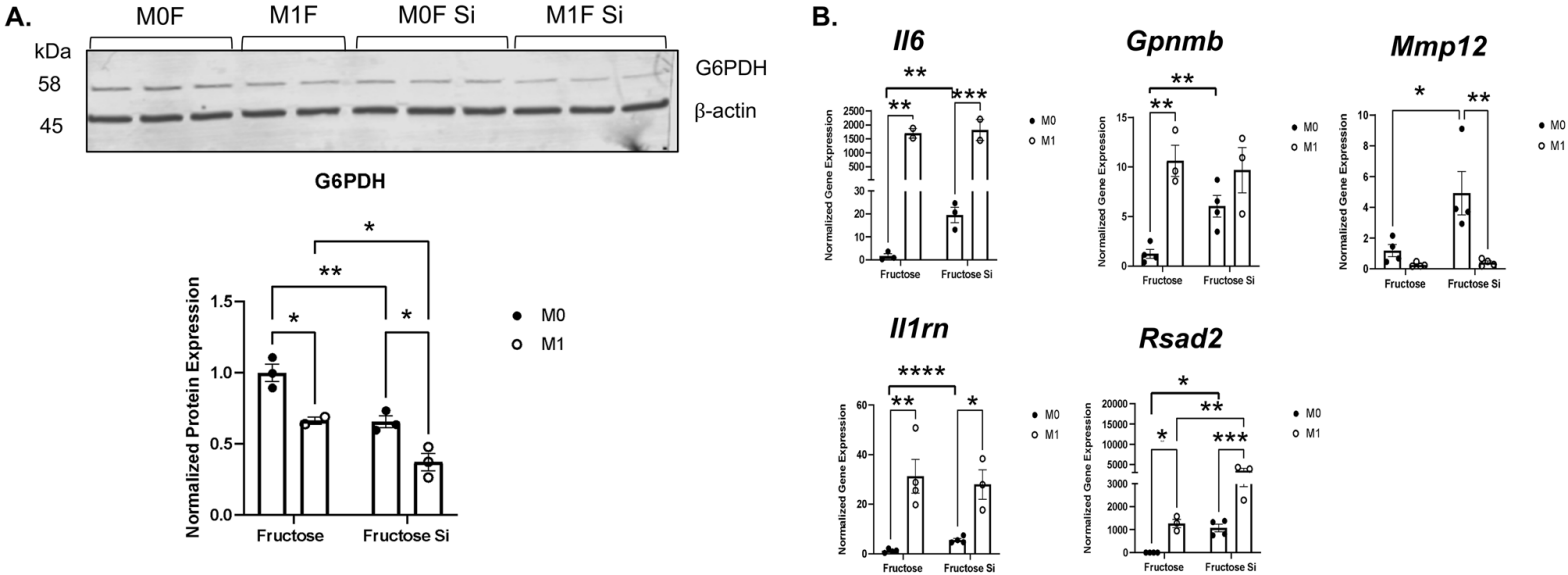
siRNA Inhibition of the PPP Increases Fructose Induced Expression of Resolution and Anti-Inflammatory Genes in M0 IMKC. IMKC were plated in 25 mM glucose for 24-h. Media was changed to 25 mM glucose with or without 25 pmol G6PDH SiRNA for 48 h. Cells were treated with 25 mM fructose without (M0) or with (M1) 0.1ug/ml LPS for 24 h. **(A)** G6PDH protein expression. The original blot is presented in Supplementary Fig. 10**(B)** Inflammatory gene *Il6* and resolution and anti-inflammatory genes *Gpnmb, Mmp12*, *Il1rn,* and *Rsad2*. Data are representative of three independent experiments and show mean ± SEMs (n = 3–4). * *P < *0.05, ** *P < *0.01, *** *P < *0.001, **** *P < *0.0001.

To specifically investigate the involvement of the PPP in the regulation of gene expression, G6PDH siRNA was used to reduce carbon shuttling through the PPP. Knockdown of G6PDH in fructose treated IMKC cells was confirmed by protein expression in M0 and M1 macrophages (Fig. 8A). Knockdown of G6PDH in fructose conditions led to increased expression of *Il6*, *Gpnmb*, *Mmp12*, *Il1rn* and *Rsad2* in M0 IMKC (Fig. 8B). In M1 polarized IMKC, knockdown of G6PDH only increased *Rsad2* expression (Fig. 8B). Similarly, G6PDH knockdown increased *Il6* gene expression in fructose treated M0 and M1 J774.1 and only increased protein expression in M1 (Supplemental Fig. 9D,E). These results suggest the PPP functions to regulate the anti-inflammatory and wound healing response of KC in the presence of fructose.

## Discussion

Investigating the impact of fructose metabolism in residential and recruited macrophages sheds new insight into how fructose feeding induces liver injury and fibrosis. Recent studies have highlighted the importance of fructose metabolism, where high concentrations of fructose bypass initial intestinal metabolism and are metabolized directly in the liver^30^. Although the literature has summarized the effects of fructose on hepatocytes, it remains unknown how fructose directly impacts the function and phenotype of macrophages^31,32^. Using fructose fed mice and in vitro macrophage cell cultures, we demonstrate that fructose metabolism directly regulates genes involved in inflammation and wound healing, cell viability, and proliferation of KC in liver tissue.

Studies of fructose feeding at different time points have yielded varying impacts on obesity, steatosis, and inflammation. Fructose exposure for 3–8 weeks increases liver steatosis without affecting body mass or liver weight^33–35^. In addition, mice had elevated infiltrating neutrophils and hepatic *Tnfα* gene expression. However, other studies show that a fructose diet increases steatosis and weight gain. Our study did not detect elevations in body weight until 20 weeks of fructose supplemented water, although liver steatosis was detected at 16 and 32 weeks. We found that 30% fructose or glucose supplementation in the water for 32 weeks increased liver and adipose tissue weights. The difference between our study and other groups may be due to the limited fat content in the chow diet and fructose delivery through the drinking water versus the diet^36^.

Several indirect mechanisms of fructose metabolism regulate hepatic macrophage function and phenotype under NASH conditions. We demonstrated that fructose increased acylcarnitines and diacylglycerides, specifically saturated long-chain fatty acids, although we only detected changes at 20 weeks of diet. A recent study reported a chow diet with 30% fructose (w/v) for 10 weeks elevated saturated acylcarnitine levels^37^. As shown in previous studies, increased acylcarnitines may indicate inhibition of fatty acid oxidation and cause mitochondria dysfunction through increased ROS^38^. In addition, elevated acylcarnitine levels can increase Tnfα expression, especially within macrophages^39^. Therefore, fructose induced acylcarnitines may drive increased inflammatory cytokine gene expression within KC of the liver.

Fructose is also reported to increase uric acid levels through mucosal microbiome and hepatic cell fructose metabolism^40^. Uric acid and oxidative stress drive inflammation in human macrophages and mouse kidneys. In fructose diet studies, TNFα protein expression and hepatic macrophage infiltration are increased in mice and dependent on oxidative stress from fructose induced intestinal leaky gut endotoxin release^41^. Interestingly, our fructose data demonstrated a significant elevation of *Tnfα* gene expression in liver tissue and IMKC, RAW 264.7 cells and J774.1 macrophages. Therefore, pro-inflammatory cytokine expression in KC may be linked to microbiome induced oxidative stress by endotoxin release, uric acid mediated inflammatory signaling and direct fructose metabolism by KC.

Fructose feeding also increased hepatic fibrotic gene expression and upregulation of inflammatory genes. These tissues also scored higher in liver injury and had elevated collagen deposition. Diet studies monitoring patients with NAFLD found that daily fructose consumption rather than high fructose consumption over short periods decreased steatosis and increased the presence of fibrosis in human liver biopsies^23^. In addition, the release of TNFα from activated KC contributes to liver fibrosis by increasing *Timp1* gene expression from hepatic stellate cells (HSCs)^42^. We previously reported that fructose increases *Timp1* expression compared to glucose in the liver and J774.1 cells^43^. Tnfα signaling deficiencies in KC are reported to attenuate liver steatosis and fibrosis, indicating a critical role of KC in the pathogenesis of NAFLD. One study demonstrates that KC depletion in the presence of marginal copper deficiency and a high fructose diet decreases steatosis and hepatic inflammation^44^. In this study, fructose significantly upregulated *Tnfα* gene expression in the liver, which may drive the upregulation of *Timp1* gene expression. Although studies have found IL6 increases in both serum and liver tissue of mice on a fructose diet, our studies did not indicate an effect of fructose on *Il6* gene expression in liver tissue^45,46^. Interestingly, the pro-fibrotic response of KC was congruent with increased gene expression of anti-inflammatory and resolution associated genes *Gpnmb*, *Mmp12*, *Il1rn* and *Rsad2* in the liver, hepatic macrophages, and IMKC. Fructose metabolism did not only induce pro-inflammatory genes but also induced genes associated with fibrosis, resolution, and anti-inflammation, supporting the plasticity of macrophages during metabolic disease.

Recent studies have found GPNMB protective in NAFLD, where overexpression of GPNMB reduces fat accumulation and fibrosis in the liver^47^. The protective effect of GPNMB may be due to its anti-oxidative properties and suppression of pro-inflammatory cytokine release. Interestingly, GPNMB increases during macrophage differentiation^9^. In this study, we show fructose increases *Gpnmb* gene expression, which may be in response to elevated transitioning monocytes. To our knowledge, this is the first report of fructose induced *Gpnmb* gene expression in macrophages. In addition, Il1RN may lead to anti-inflammatory properties as the protein blocks the inflammatory signaling of IL-1β. Along with the upregulation of *Il1rn* gene expression in IMKC, fructose also decreased *Il1b* gene expression. The combined effect of these responses may indicate that the PPP suppresses the macrophage anti-inflammatory response in the presence of fructose based on the knockdown of G6PDH, increasing anti-inflammatory and wound healing gene expression.

In liver injury, KC secrete MMPs to aid in fibrosis regression and tissue damage resolution^48^. In addition, MMP12 is pro-inflammatory and necessary for wound healing^49^. Recent studies suggest that the absence of MMP12 attenuates leaky gut and reduces immune cell infiltration and transmigration across the colonic epithelial barrier, leading to decreased inflammation and disease severity^49,50^. MMP12 is also necessary for fibrosis resolution, as inhibition of MMP12 increases elastin accumulation and fibrosis^51^. We found that fructose directly increases *Mmp12* gene expression in the liver and IMKC. In addition, *Rsad2* expression increases in fructose treated IMKC, which can directly regulate inflammatory interferon signaling as knockdown dampens type I interferon and reduces T cell activation^52^. Interestingly, MMP12 may also stimulate pro-inflammatory cytokine expression and regulate macrophage proliferation^53^. Fructose increased *Mmp12* can alter monocyte proliferation or increase inflammatory cytokines for tissue clearance and wound healing. Fructose may directly reduce KC and induce signals responsible for monocyte recruitment. The increase in transition monocytes may result from reduced viability of KC and push the liver to recruit monocytes. However, fructose may directly inhibit the ability of recruited monocytes to differentiate into macrophages to replenish KC. Likewise, fructose may impair KC proliferation. However, we found that fructose increased IMKC proliferation but did not detect differences in proliferating macrophage gene signatures between glucose or fructose fed mice.

C13 fructose tracing revealed novel insights into fructose metabolism in IMKC, RAW 264.7 and J774.1 cells. Glucose transporters GLUT2 and 8 are known to transport fructose within the liver. However, macrophages primarily express transporters GLUT 1 and 3^54–56^. A recent paper indicated the presence of GLUT5 within bone marrow derived macrophages, although this mechanism may differ between macrophage subsets^13^. Liang, R.J. et al. reported that multiple cancer cell lines do not metabolize fructose, often leading to decreased proliferation and cell death ^57^. However, overexpression of GLUT5 increases cell proliferation and survival under fructose conditions, indicating a potential for energy source plasticity and a regulatory role of GLUT transporters over HKs. This may occur in KC where fructose exposure may upregulate fructose transporters. Fructose is phosphorylated by KHK, which is expressed in liver, kidneys, and pancreas. We analyzed KHK expression in IMKC and liver myeloid cells from mice fed glucose or fructose and found no expression of KHK. HK1 phosphorylates glucose and fructose in macrophages^58^. Although glucose is the preferred substrate of HK1, concentrations of 5 mM or more of fructose increase the reaction rate to be equal to glucose^59^.

Fructose metabolism utilizes fructose -1- phosphate, which links fructolysis with glycolysis^60^. Given the connection between glycolysis and the TCA cycle, it is interesting that glycolytic intermediates were detected in all cell lines, where TCA cycle intermediates were very low. Jones et al. reported human peripheral blood monocytes treated with 11.1 mM C13 fructose induced fructose carbon flux through the TCA cycle^12^. This cycling was accompanied by increased secretion of inflammatory proteins. LPS stimulation of macrophages upregulates glycolysis and the PPP to supply energy and intermediates for increased inflammatory cytokine production^15,61^. M1 polarization drives inflammation through increased ROS production from glycolysis and PPP intermediates through LPS stimulated TLR4 activation. However, M2 polarized macrophages use the TCA and OXPHOS to supply ETC with intermediates needed for proliferation. Here, we demonstrate that fructose drives macrophage plasticity as fructose tracing reveals carbon shuttling into glycolysis and the PPP without polarization with LPS. The PPP regulates inflammation by producing NADPH for oxidant and antioxidant properties for macrophage clearance of pathogens. Inhibition of the PPP increased gene expression of both pro and anti-inflammatory genes. Fructose challenges IMKC inflammatory regulation by activating glycolysis and the PPP and decreasing TCA shuttling. The lack of succinate in fructose samples may be responsible for reduced *Il1b* gene expression compared to glucose. Our results indicate a preference for fructose carbon shuttling through the PPP.

The differences between metabolic partitioning may be due to cell type and fructose concentration. Inhibition of the PPP using 6AN decreases LPS induced high fat diet macrophage inflammatory cytokine expression^29^. LPS induces metabolic reprogramming by increasing glycolysis and the PPP. Similar to this study, our results indicate decreased *Gpnmb*, *Il6* and *Il1rn* expression in fructose treated M1 IMKC treated with 6AN. Unique to our study, fructose stimulated the expression of *Gpnmb*, *Mmp12* and *Il1rn*. PPP inhibition by G6PDH knockdown significantly increased the expression of *Il6*, *Gpnmb*, *Mmp12*, *Il1rn* and *Rsad2*. The significance of our data demonstrates that fructose causes resident and differentiated macrophages to adopt a wound healing and anti-inflammatory phenotype, which leads to reduced viability. Inhibition of the PPP leads to increased expression of genes in both pathways. Our findings indicate that fructose may leave KC metabolically impaired, trying to regulate survival, proliferation, and inflammatory response to the environment driving a wound repair phenotype. While fructose increases the anti-inflammatory response of KC, shuttling fructose carbons through the PPP may decrease their potential to aid in wound repair and healing efficiently, leaving the tissue fibrotic and inflamed.

In conclusion, chronic fructose exposure causes liver injury and increased anti-inflammatory and resolution associated genes in KC. Fructose metabolism within KC use glycolysis and the PPP. KC are equipped to handle alternate carbohydrate sources as they must adapt quickly to respond to surrounding stimuli. Interestingly, this response was coupled with reduced KC while increasing transitioning monocytes. In vitro studies indicate that the PPP suppresses fructose regulation of anti-inflammation and resolution genes.

### Supplementary Information

Supplementary Legends.

Supplementary Figures.

## Data Availability

The original data presented in the manuscript are included in the article. The datasets generated during and analyzed are available from the corresponding author on reasonable request.
