# Supplementary material for: Fructose regulates the pentose phosphate pathway and induces an inflammatory and resolution phenotype in Kupffer cells
Source: Sci Rep. 2024 Feb 18;14:4020. doi: 10.1038/s41598-024-54272-w (PMC10874942; doi:10.1038/s41598-024-54272-w)
Supplement: Supplementary file 1 — Supplementary Legends. [file 41598_2024_54272_MOESM1_ESM.pdf]

Supplemental Figure Legend

Supplemental Figure 1. Metabolic and Inflammation in Fructose fed C57BL/6 Mice at 16 Weeks

C57BL/6 mice were on chow diet with 30% glucose or fructose for 16 weeks. Liver and adipose tissue were removed and weighed at 16 weeks **(A,B).** Liver tissue was harvested for RT qPCR to analyze *Fasn*, *Tnfa, Col1a1,* and *Timp1* (N=5-6) **(C)** and tissue was sectioned and isolated for mass spectrometry analysis of total triglycerides and diglycerides **(G).** At 16 and 32 weeks, GPT2 protein levels were measured by western blot analysis **(H)** Original blots in Supplemental Figure 2**.** Data are representative of 3 independent experiments and shown as means ± SEMs.* $p<0.05$, ** $p<0.01$, *** $p<0.001$, **** $p<0.0001$.

Supplemental Figure 2. Original Western Blot of GPT2 at 16 and 32 Weeks.

(A) GPT2 and β-actin protein expression at 16 weeks. **(B)** GPT2 and β-actin protein expression at 32 weeks.

Supplemental Figure 3. Chronic Fructose Diet Macrophage Subclusters and Gene Signatures

Dot plot generated from unbiased differential expression using a Wilcox test to identify markers for each subcluster in chronic diet studies.

Supplemental Figure 4. Fructose Uptake in J774.1 Causes Decreased Viability

RAW **(A)** and J774.1 **(B)** cells were treated with 5mM fructose for 24 hours with and without 10ng/ml LPS. Cells were harvested using MetOH extraction for mass spectrometry (n=3). **(C)** MTT analysis of J774.1 cells treated 5mM glucose or fructose with or without LPS (n=5). **(D,E)** Viability assay and cytotoxicity assay (n=6). (**F)** Measured luminescence for apoptosis assay (n=6) **G:** Cells were treated with 5mM glucose or fructose with BrdU for 24 hours before antibody labelling and reading of colorimetric absorbance (n=4). Data are representative of 3 independent experiments and shown as means ± SEMs.* $p<0.05$, *** $p<0.001$, **** $p<0.0001$.

Supplemental Figure 5. Original Western Blot of HK1 in IMKC.

(A) HK1 and β-actin protein expression and quantification Experiment 1. **(B)** HK1 and β-actin protein expression and quantification Experiment 2.

Supplemental Figure 6. Fructose Regulates Inflammatory Gene Expression in RAW and J774.1 Cells

Cells were treated with 5mM glucose or fructose for 24-hours and supplemented with/without LPS at 10 ng/mL. cDNA was generated from RNA isolation and RT qPCR was analyzed for gene expression of pro-inflammatory cytokines *Tnfα* **(A)**, *Il6* **(B)** and *Il-1b* **(C)** and anti-inflammatory gene *Gpnmb* **(D)** in RAW cells as well as *Tnfα* **(E)**, *Il6* **(F)*,*** and IL6 protein secretion measured via ELISA **(G).** Additionally, expression of *Il-1b* **(H)** and *Gpnmb* **(I)** were also measured in J774.1 cells. **I:** in J774.1. (n=4). Data are representative of 3 independent experiments and shown as means ± SEMs.* $p<0.05$, ** $p<0.01$, *** $p<0.001$, **** $p<0.0001$.

Supplemental Figure 7. Fructose Metabolism Partitioning in RAW and J774.1 Cells

Cells were plated in 5mM glucose for 24 hours before media was changed to C13 5mM glucose or fructose with 10 ng/mL LPS for M1 cells. 24 hours later cells were harvested for LC/MS via MetOH extraction for metabolite analysis and analyzed by MS peak intensity. Metabolites of interest in RAW cells were **(A-B)** glycolysis intermediates, **(C)** TCA cycle Intermediates, and D: PPP intermediates**.** For J774.1 cells, **(E-F)** Glycolysis intermediates, **(G-H)** TCA cycle

intermediates and **I:** PPP intermediates. (n=3). Data are representative of 3 independent experiments and shown as means ± SEMs.

Supplemental Figure 8. Pharmacological Inhibition of the PPP Increases Fructose Induced Expression of Anti-Inflammatory Genes in M0 IMKC

IMKCs were treated with 25mM glucose or fructose for 24-hours and supplemented with/without LPS at 0.1ug/mL. Cells were treated with 100uM 6AN at time of treatment. Cells were isolated for RNA expression and genes of inflammation, **(A-B)** *Il6* and *Tnfα* and anti-inflammatory genes **(C-F)** *Gpnmb*, *Mmp12*, *Il1rn* and *Rsad2*. (n=4). Data are representative of 3 independent experiments and shown as means ± SEMs. * p<0.1, ** p<0.01, *** p<0.001, **** p<0.0001.

Supplemental Figure 9. Pharmacological Inhibition of the PPP Increases IL6 Gene Expression in Fructose Treated J774.1 Cells.

J774.1 cells were treated with 5mM fructose with or without 10, 25 or 50 μM DHEA for 24 hours as well as with or without 10ng/ml LPS. Cells were isolated for RNA and analyzed by RT qPCR inflammatory genes *Il6*, **(A)** and *Tnfα* **(B)** and for fibrotic gene *Timp1* **(C). (D**) SiRNA knockdown was performed by adding 25pmol G6PDH SiRNA with lipofectamine for 48 hours in 5mM fructose conditions and analyzed by RT qPCR for *Il6* expression. **(E)** SiRNA treatment media was kept and analyzed by ELISA for IL6 protein secretion. (n=4-5). Data are representative of 2-3 independent experiments and shown as means ± SEMs. ** p<0.01, *** p<0.001, **** p<0.0001.

Supplemental Figure 10. Original Western Blot of G6PDH in IMKC.
