# Supplementary material for: Fructose regulates the pentose phosphate pathway and induces an inflammatory and resolution phenotype in Kupffer cells
Source: Sci Rep. 2024 Feb 18;14:4020. doi: 10.1038/s41598-024-54272-w (PMC10874942; doi:10.1038/s41598-024-54272-w)
Supplement: Supplementary file 2 — Supplementary Figures. [file 41598_2024_54272_MOESM2_ESM.pdf]

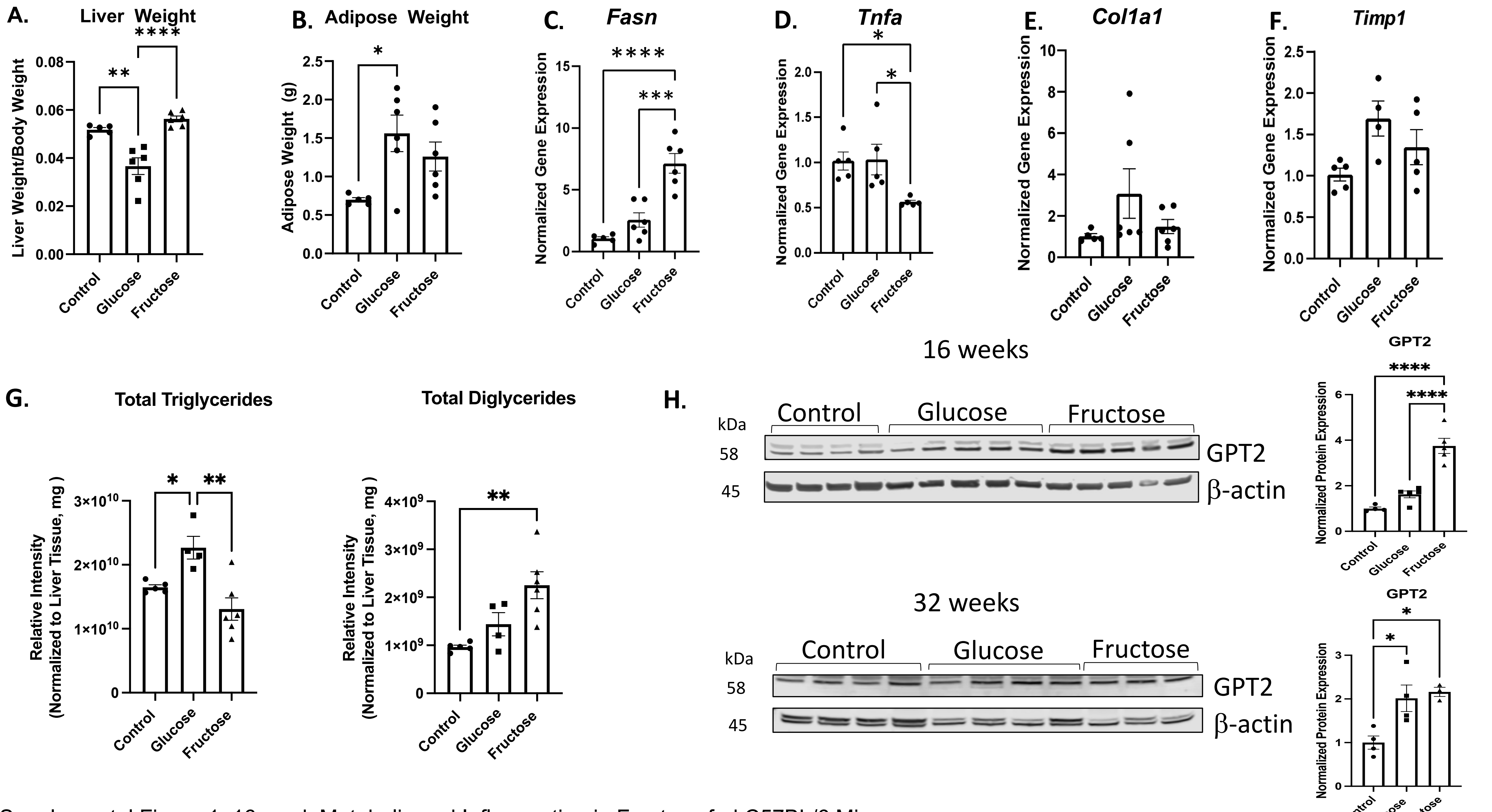

Supplemental Figure 1. 16 week Metabolic and Inflammation in Fructose fed C57BL/6 Mice

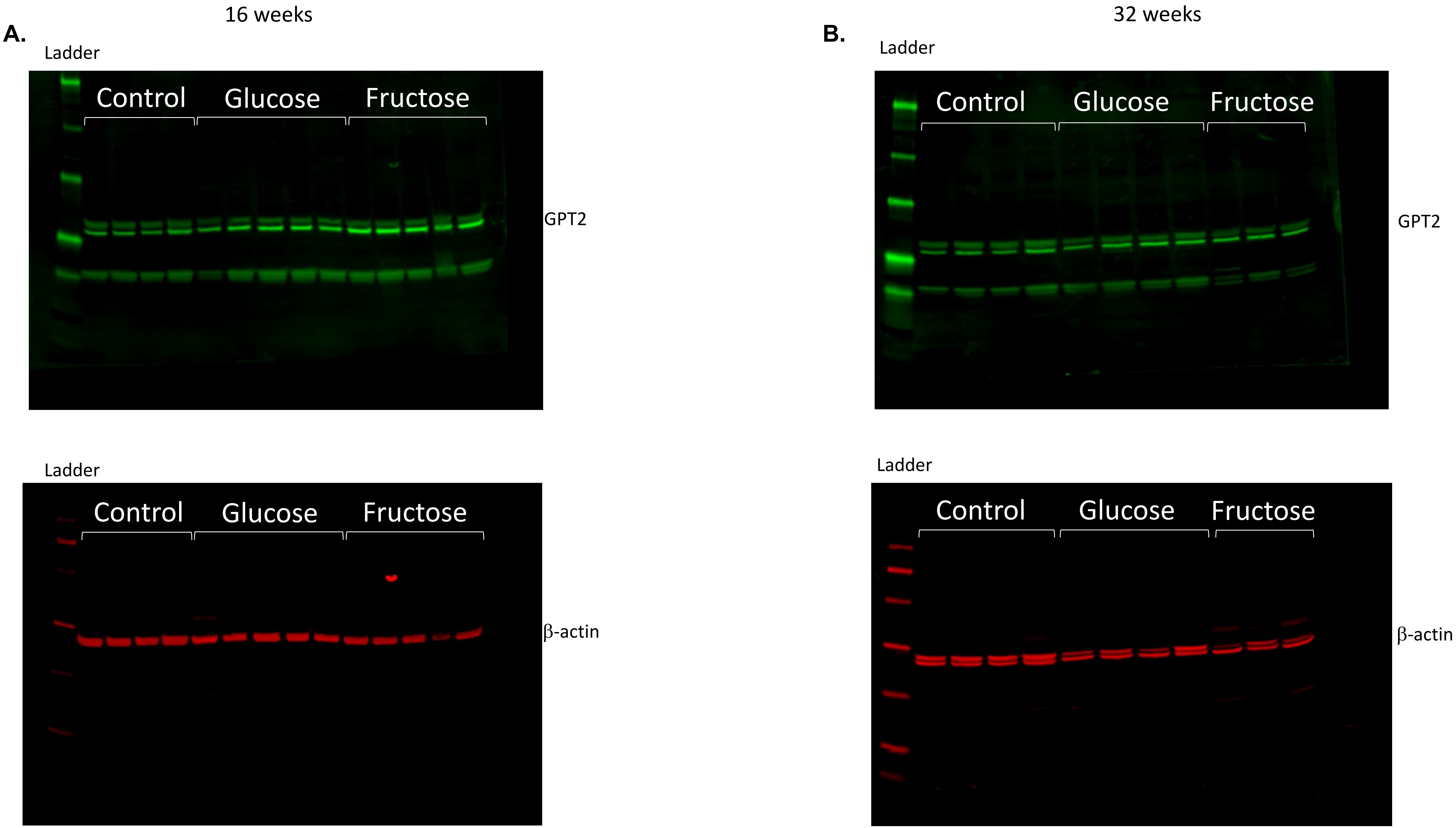

Supplemental Figure 2. Original Western blots of GPT2 and b-actin

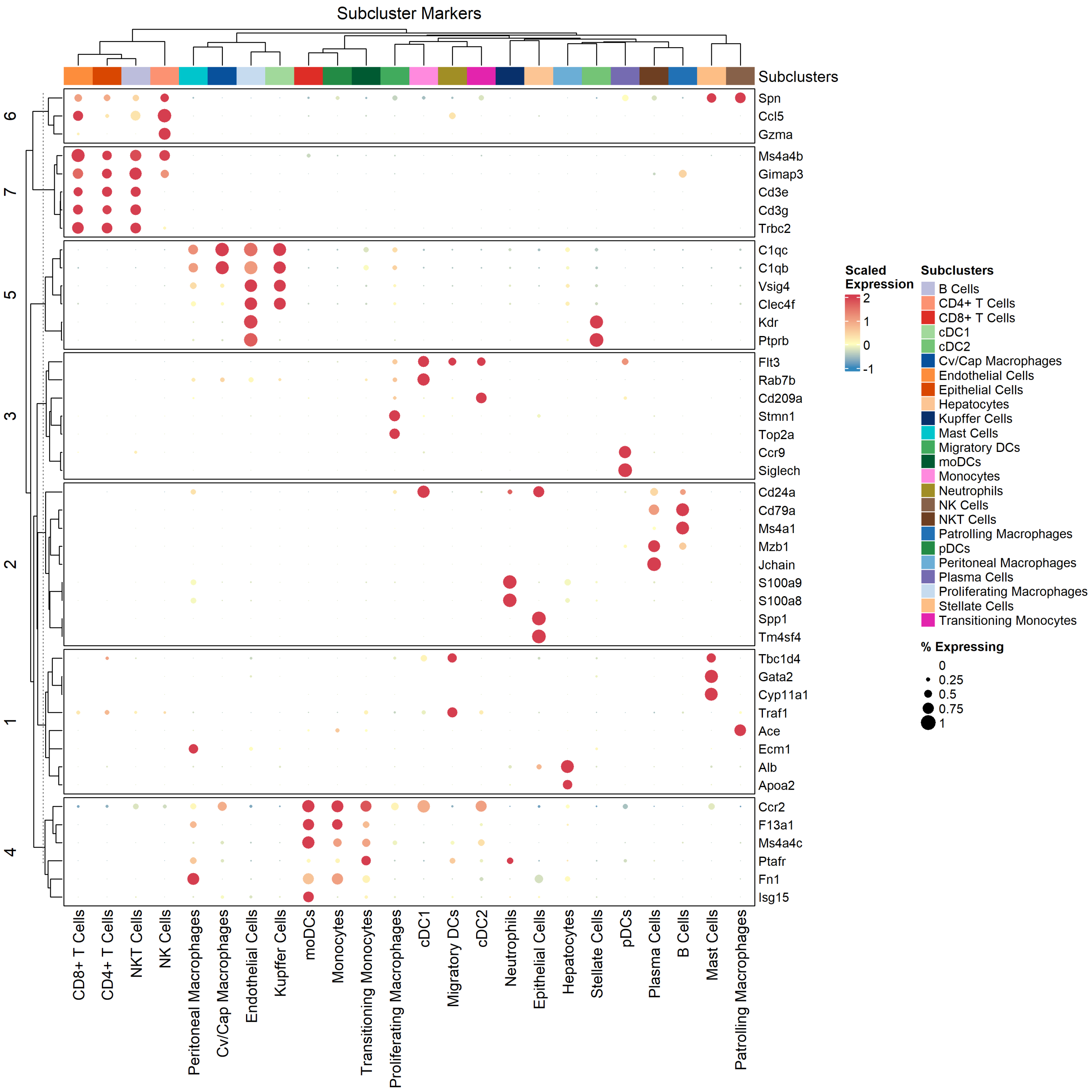

Supplemental Figure 3. Chronic Fructose Diet Macrophage Subclusters and Gene Signatures

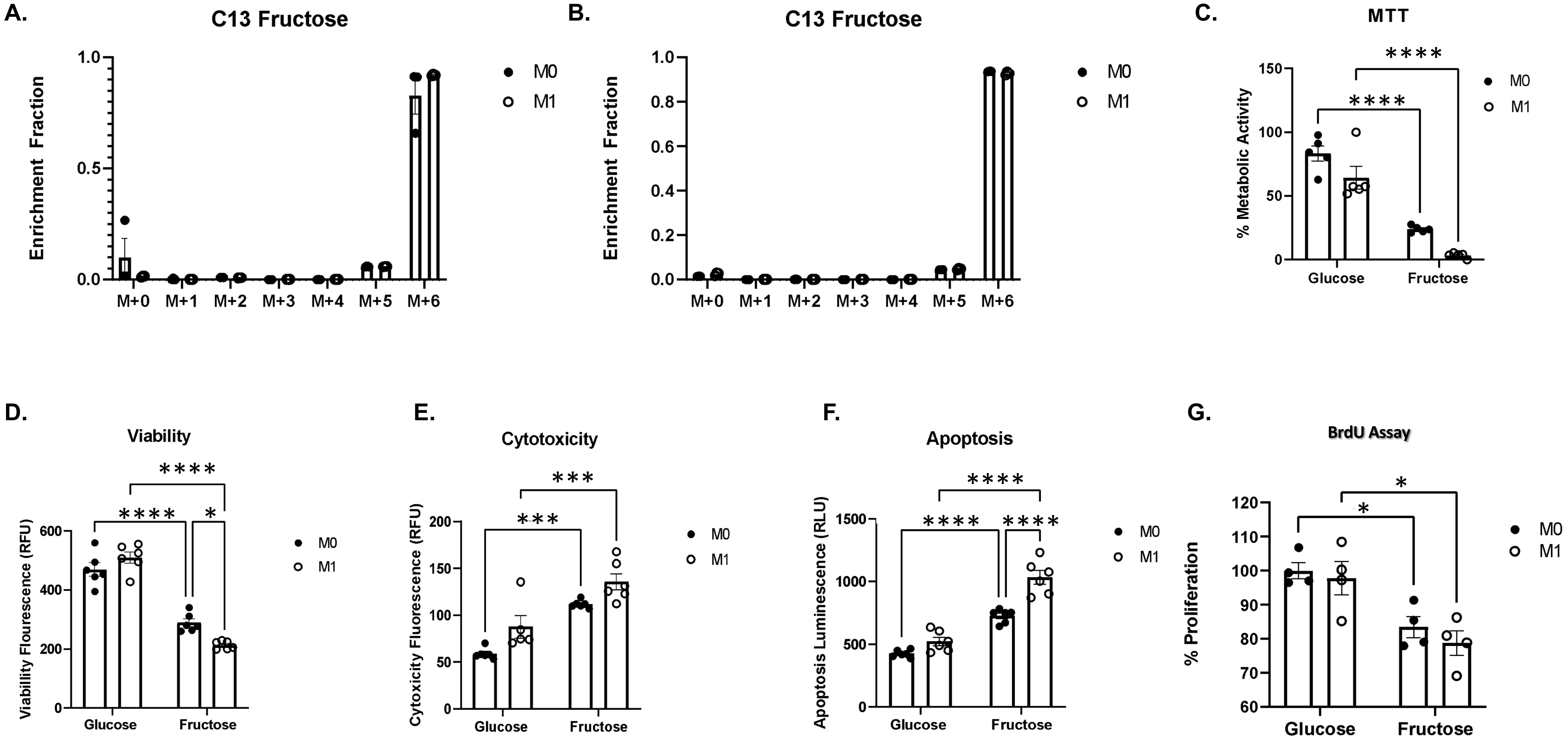

Supplemental Figure 4. Fructose Uptake In J774.1 Causes Decreased Viability

A.

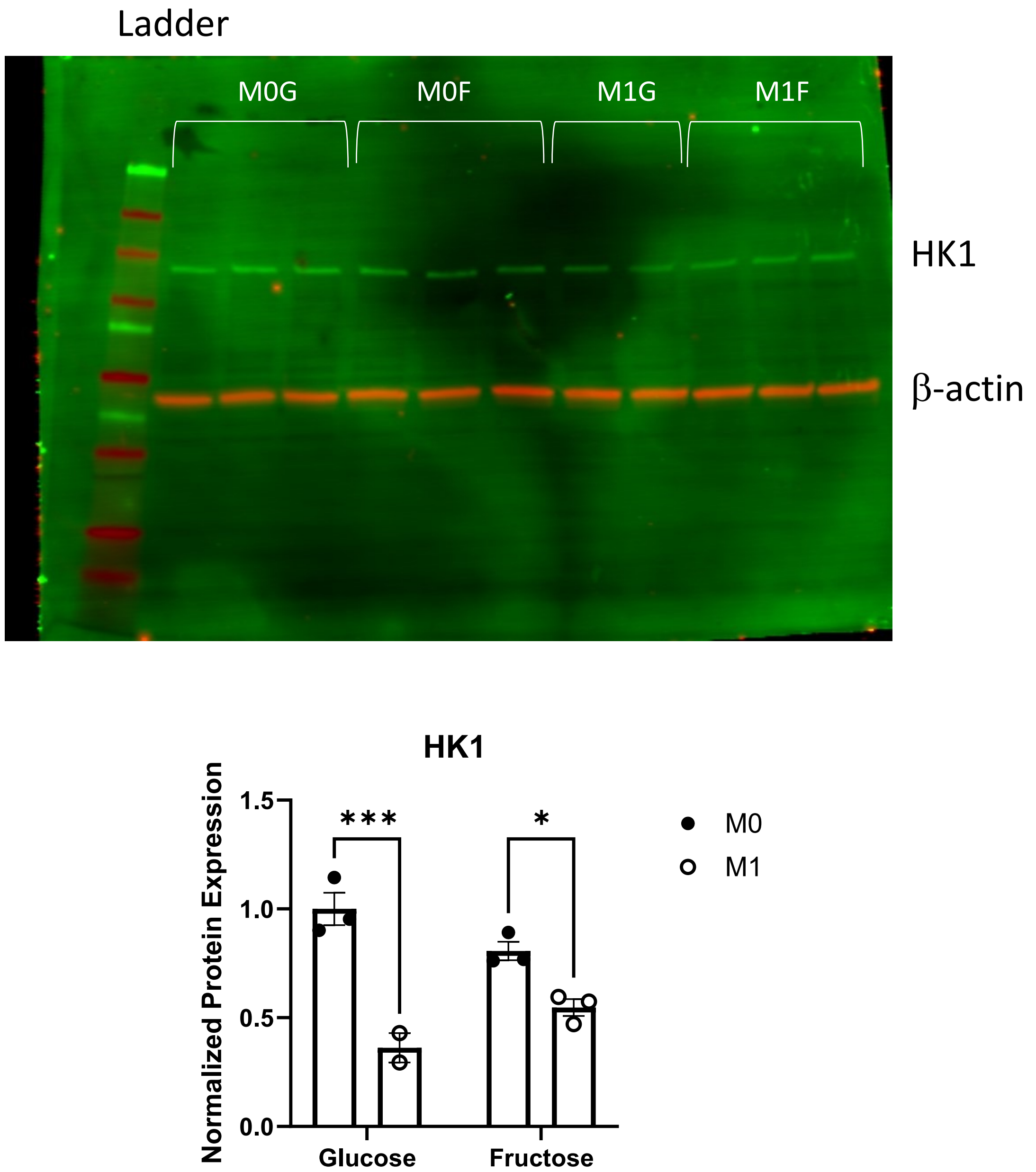

B.

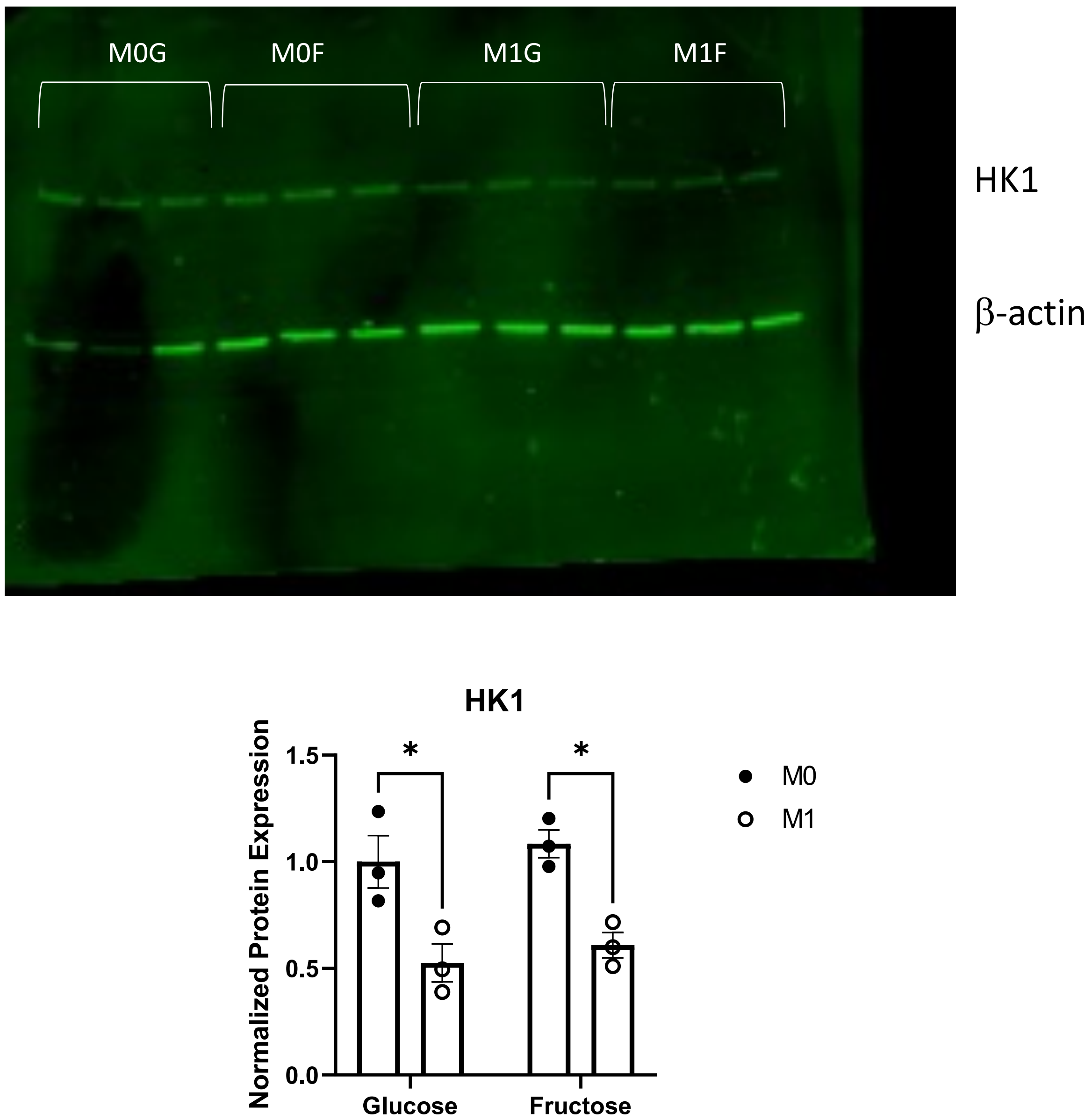

Supplemental Figure 5. Original Western blots of HK1 and b-actin

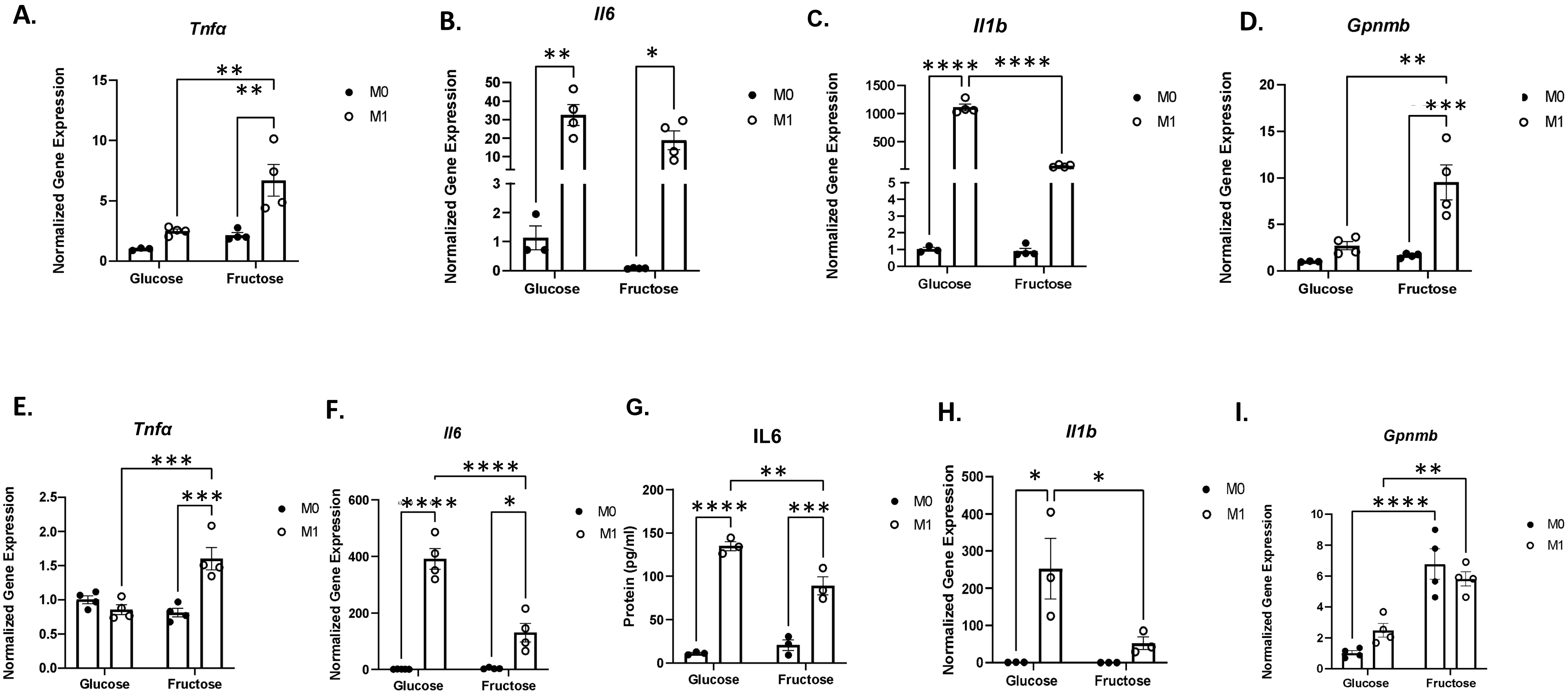

Supplemental Figure 6. Fructose Regulates Inflammatory Gene Expression in RAW and J774.1 Cells

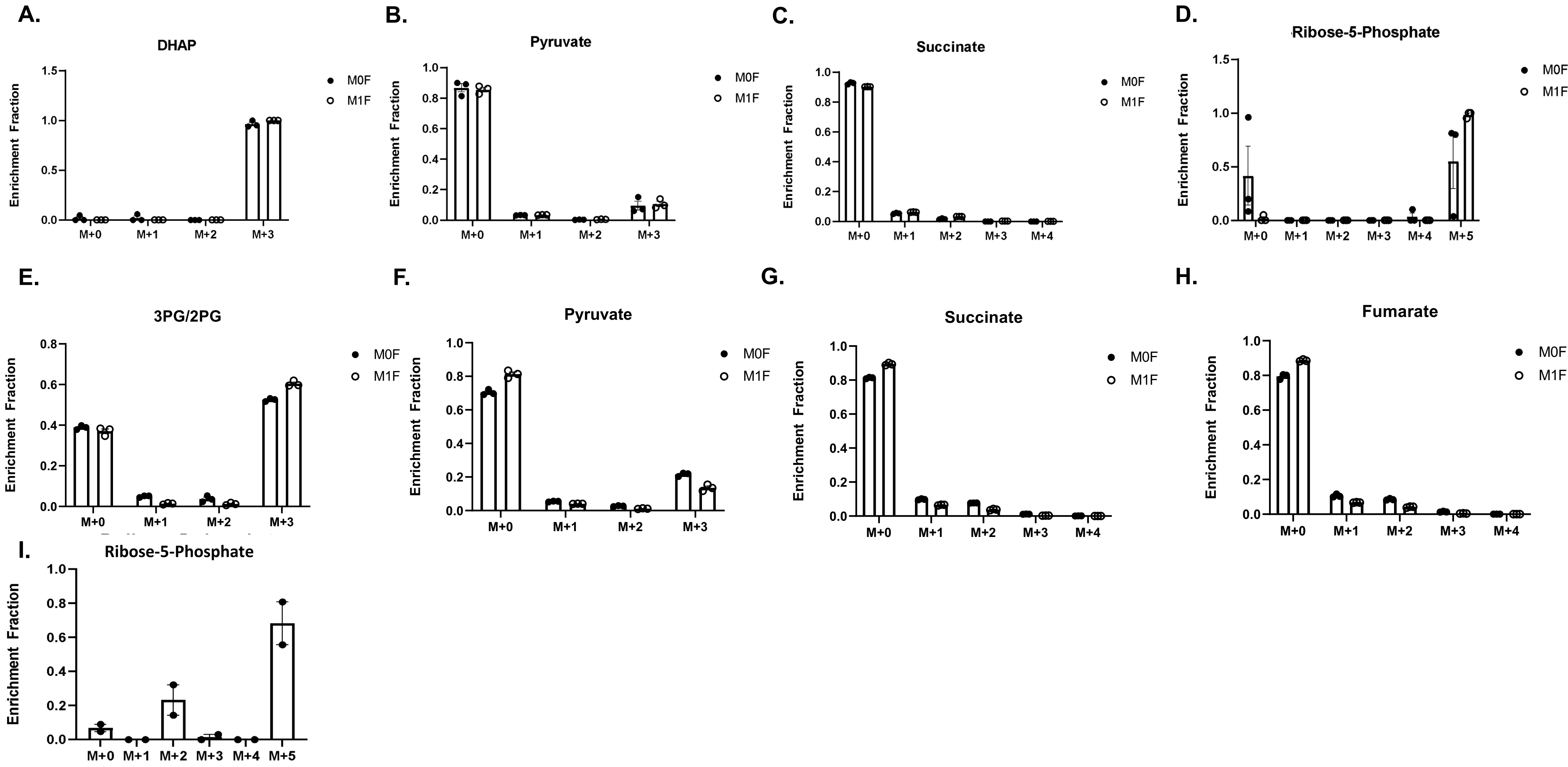

Supplemental Figure 7. Fructose Metabolism Partitioning Within RAW and J774.1 Cells

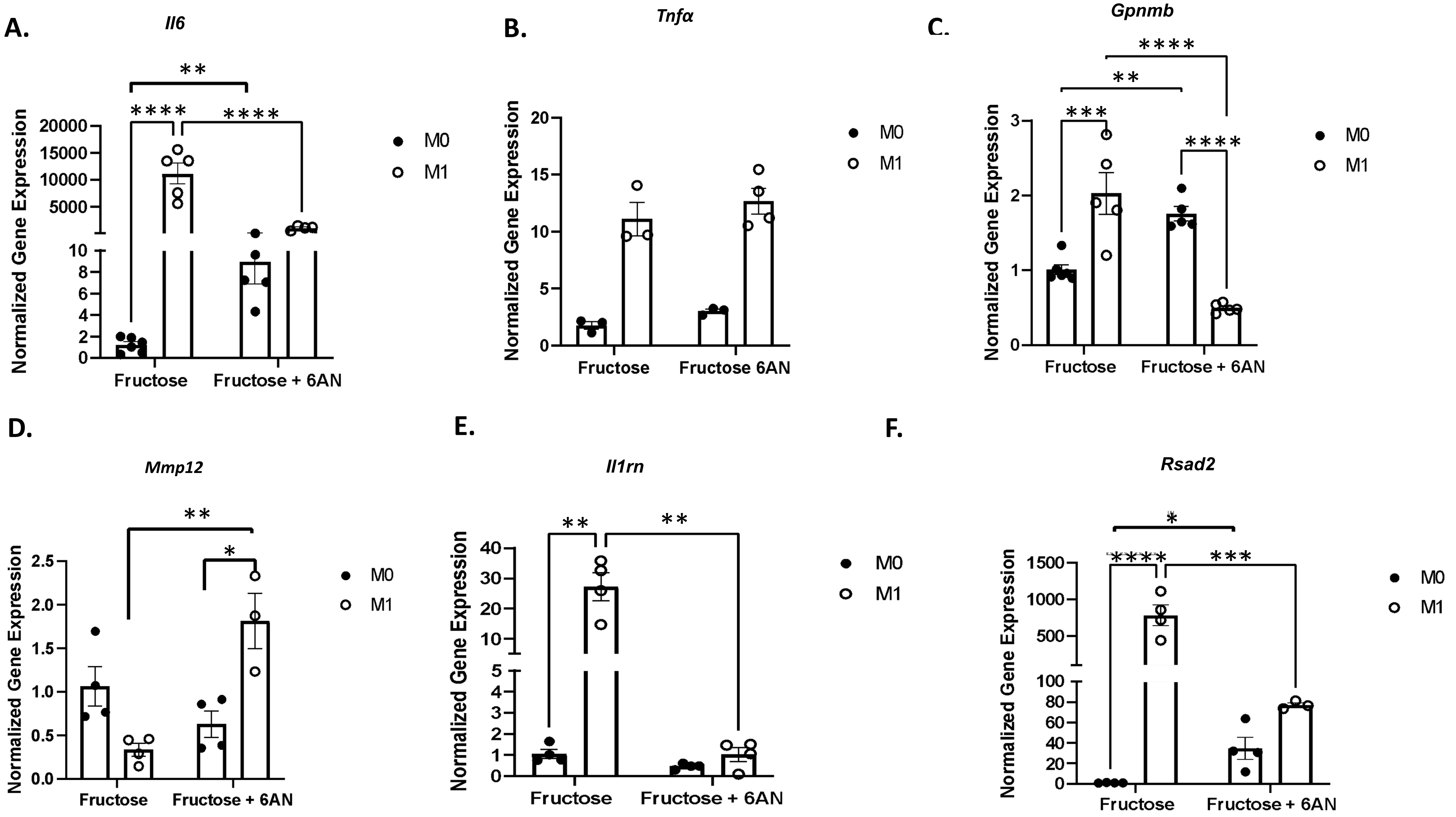

Supplemental Figure 8. Pharmacological Inhibition of the PPP Increases Fructose Induced Expression of Anti-Inflammatory Genes in M0 IMKC

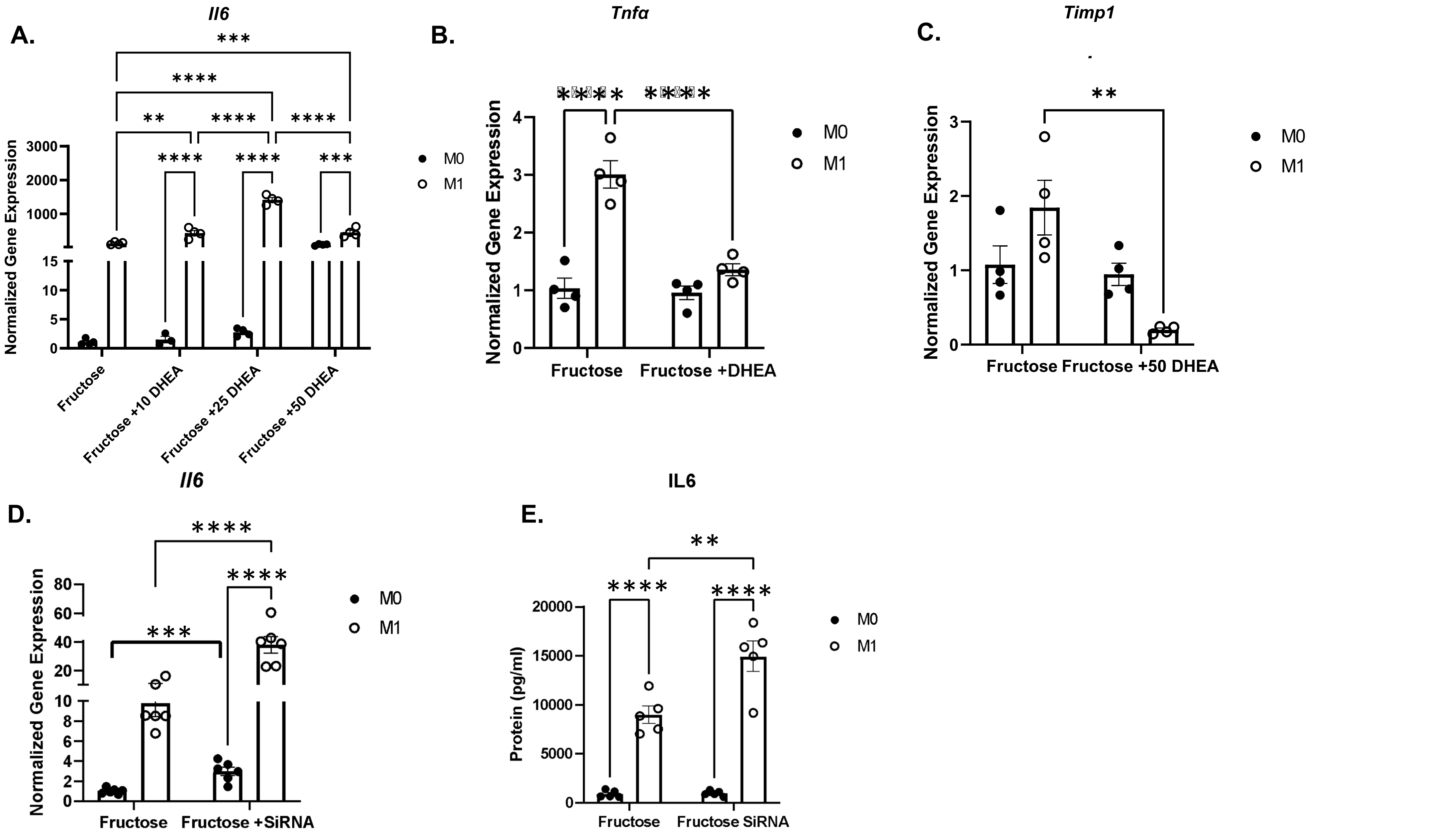

Supplemental Figure 9. Pharmacological Inhibition of the PPP Increases IL6 Gene Expression in Fructose Conditions

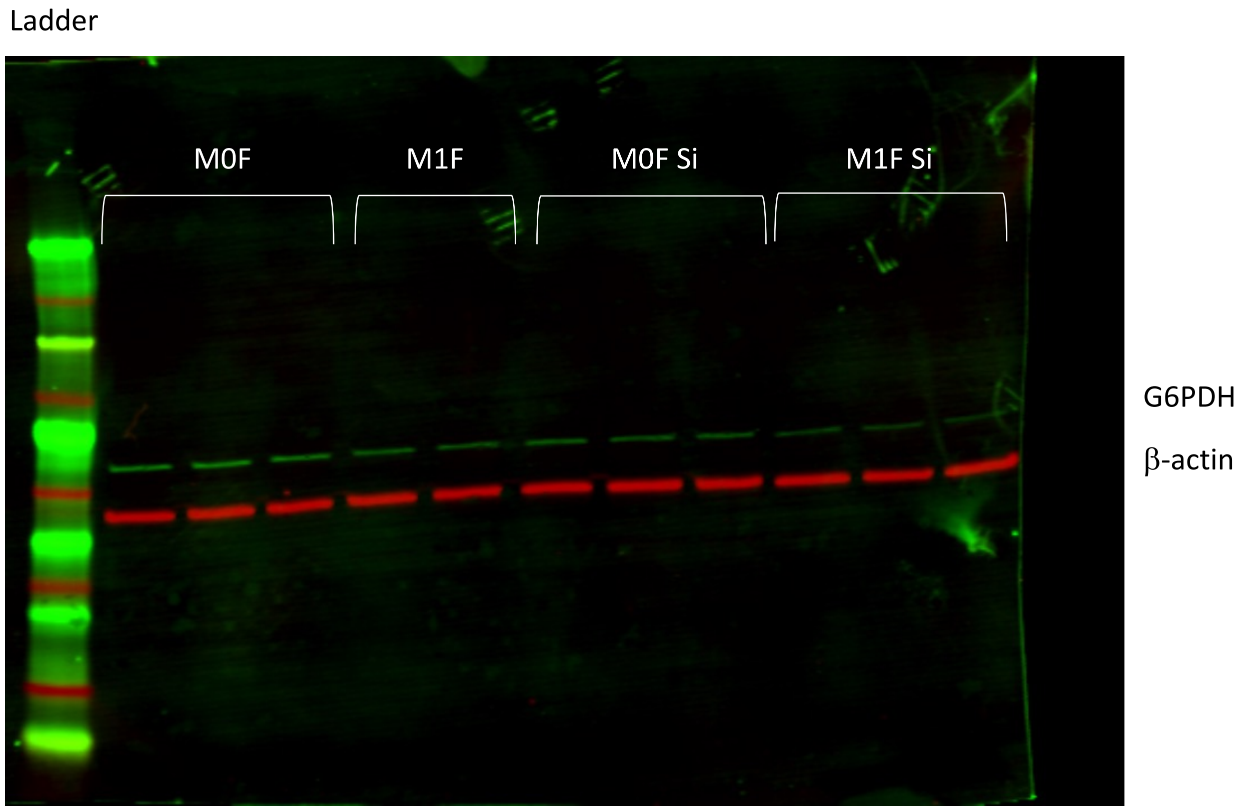

Supplemental Figure 10. Original Western blot of G6PDH and b-actin
